# Associations between primary care recorded cannabis use and mental ill health in the UK: a population-based retrospective cohort study using UK primary care data

**DOI:** 10.1017/S003329172100386X

**Published:** 2023-04

**Authors:** Deepiksana Keerthy, Joht Singh Chandan, Juste Abramovaite, Krishna Margadhamane Gokhale, Siddhartha Bandyopadhyay, Ed Day, Steven Marwaha, Matthew R. Broome, Krishnarajah Nirantharakumar, Clara Humpston

**Affiliations:** 1 Institute of Applied Health Research, College of Medical and Dental Sciences, University of Birmingham, Birmingham B15 2TT, UK; 2 Institute of Global Innovation, University of Birmingham, Birmingham B15 2TT, UK; 3 The Department of Economics, University of Birmingham, Birmingham B15 2TT, UK; 4 Institute for Mental Health, College of Life and Environmental Sciences, University of Birmingham, Birmingham B15 2TT, UK; 5 Birmingham and Solihull Mental Health NHS Trust, Birmingham B1 3RB, UK; 6 Birmingham Women' and Children' NHS Foundation Trust, Birmingham B15 2TG, UK

**Keywords:** Anxiety, bipolar disorder, cannabis use, depression, epidemiology, schizophrenia

## Abstract

**Background:**

Cannabis use is a global public health issue associated with increased risks of developing mental health disorders, especially in young people. We aimed to investigate the relationships between cannabis exposure and risks of receiving mental illness diagnoses or treatment as outcomes.

**Methods:**

A population based, retrospective, open cohort study using patients recorded in ‘IQVIA medical research data’, a UK primary care database. Read codes were used to confirm patients with recorded exposure to cannabis use who were matched up to two unexposed patients. We examined the risk of developing three categories of mental ill health: depression, anxiety or serious mental illness (SMI).

**Results:**

At study entry, the exposed cohort had an increased likelihood of having experienced mental ill health [odds ratio (OR) 4.13; 95% confidence interval (CI) 3.99–4.27] and mental ill health-related prescription (OR 2.95; 95% CI 2.86–3.05) compared to the unexposed group. During the study period we found that exposure to cannabis was associated with an increased risk of developing any mental disorder [adjusted hazard ratio (aHR) 2.73; 95% CI 2.59–2.88], also noted when examining by subtype of disorder: anxiety (aHR 2.46; 95% CI 2.29–2.64), depression (aHR 2.34; 95% CI 2.20–2.49) and SMI (aHR 6.41; 95% CI 5.42–7.57). These results remained robust in sensitivity analyses.

**Conclusion:**

These findings point to the potential need for a public health approach to the management of people misusing cannabis. However, there is a gross under-recording of cannabis use in GP records, as seen by the prevalence of recorded cannabis exposure substantially lower than self-reported survey records.

## Background

Despite the legal status of cannabis use in most countries, the prevalence of recreational use of cannabis and cannabis-containing products have been increasing in a number of developed countries (including the United States, Australia and New Zealand) whereas figures suggest a decline in other countries (including the UK) over the past two decades (Degenhardt et al., 2013; Hall et al., 2019; Peacock et al., 2018). Compared to other substances of misuse, cannabis has been considered to be one of the ‘safer’ drugs (deemed by the ratio of toxicological threshold in comparison to human intake), and has shown promise in medical therapies leading to movements for legalisation globally (Hall et al., 2019; Lachenmeier & Rehm, 2015). Although there are suggestions that cannabis appears to have fewer harmful effects to people than other substances, numerous studies have pointed to the associations between cannabis use and mental ill health, particularly serious mental illnesses (SMI; schizophrenia, bipolar disorder and other psychoses). This association has been shown to be mediated by the chemical composition of the drug used. Cannabis products containing a high level of tetrahydrocannabinol (THC) appear to have a greater damaging health effect than other formulations rich in cannabidiol (CBD). In a recent systematic review and meta-analysis of trials whereby participants are exposed to cannabis-related compounds and compared to other unexposed healthy individuals, it was shown that a single dose of THC was able to induce pronounced schizophrenia-like symptoms in healthy volunteers (Hindley et al., 2020).

Numerous systematic reviews of observational studies have demonstrated that prolonged cannabis use and misuse have been associated with a range of mental health problems including; psychosis, depression, mania, anxiety, personality disorder-related traits, suicidal and self-injurious behaviours and impulsivity (Gobbi et al., 2019; Lowe, Sasiadek, Coles, & George, 2019; Marwaha, Winsper, Bebbington, & Smith, 2018; Moore et al., 2007; Sideli, Quigley, La Cascia, & Murray, 2020). Moore et al. (2007) demonstrated in a systematic review and meta-analysis of 35 longitudinal studies that there was an increased risk of any psychotic outcomes in individuals who had ever used cannabis [pooled adjusted odds ratio (aOR) = 1.41, 95% confidence interval (CI) 1.20–1.65], and also identified a clear dose−response relationship in individuals exposed to higher doses of cannabis (aOR 2.09; 95% CI 1.54–2.84). Interestingly, in that review, when examining subsequent affective disorder outcomes, the associations were inconsistent amongst the studies included, which is consistent with other national observational studies and more recent systematic reviews (Degenhardt, Hall, & Lynskey, 2001; Gobbi et al., 2019; Sideli et al., 2020). Another recent cohort study by Hines and colleagues, in contrast, found high potency cannabis to be more closely related to risks of developing anxiety disorders (aOR 1.92; 95% CI 1.11–3.32) rather than psychotic disorders (aOR 1.29; 95% CI 0.67–2.50) in terms of mental health outcomes in adolescence (Hines et al., 2020). However, a further meta-analysis of previous reviews (‘a review of reviews’) focusing on psychosis outcomes found a dose-dependent relationship between risks of psychosis and cannabis use (Hasan et al., 2020).

When examining the current evidence base, it is clear that limitations exist which may be responsible for the inconsistent findings. Current observational studies included in the relevant reviews are prone to recall bias and social desirability bias (which could act in either direction), due to self-reporting and often the majority of the data were derived from case–control studies. Social desirability bias for example could act in both directions: under-reporting of cannabis use because of its illicit status or over-reporting because of trying to please the experimenter. This has proven to be an issue particularly in studies where survey responses are used to measure substance misuse (Johnson & Fendrich, 2005). Although causality has been explored between cannabis use and SMI (Vaucher et al., 2018), substantial concerns exist around reverse causality or a third factor causing both mental ill health and cannabis use (e.g. mental ill health predicting use of cannabis, and social adversity predicting both mental ill health and use of cannabis) and lack of control for appropriate confounders (such as genetic risk, alcohol use, co-existing tobacco use, socio-demographic factors such as deprivation and other illicit drug use) which may be responsible for the relationship between cannabis use and depression or anxiety (Sideli et al., 2020). Additionally, the majority of epidemiological evidence comes from North America and may not be generalisable to a UK population due to differences in demographic structure, views on cannabis use, access to healthcare services, criminal justice framework and extent of the welfare state. Lastly, we could not identify any studies which have not only explored the diagnosis of mental ill health but also explored the need for incident pharmacological therapies. The latter served as a proxy for diagnosed disorders so there would be a higher likelihood of accurate outcome validation if an individual scored positive on both formal diagnosis and pharmacological therapy.

Due to existing limitations in the literature, the impact of cannabis must urgently be explored in non-American cohorts and in particular the UK, where up to one third of all adults have tried cannabis (Office of National Statistics, 2018). This work also has international importance where effects of cannabis exposure may be compared between countries, which can only be reliably achieved by large cohort studies. Therefore, we have conducted the first retrospective cohort study using the IQVIA medical research data UK (IMRD-UK) database (previously named ‘The Health Improvement Network (THIN)’ database) to investigate the relationships between cannabis exposure and the development of subsequent mental ill health (defined through both diagnostic codes for depression, anxiety and SMI as well as initiation of medication) after accounting for important confounders and pre-existing mental ill health. The hypotheses for the current study were that General Practitioner (GP) recorded cannabis use at baseline would be significantly associated with the development of mental ill health, in particular cases of SMI such as psychotic disorders.

## Methods

### Study design, population and participants

This study is a population based, retrospective open cohort study using the IMRD-UK database, set between 1^st^ January 1995 and 31st December 2018. IMRD-UK consists of primary care records, deemed representative of the national population in terms of demographic structure and prevalence of key comorbidities (Blak, Thompson, Dattani, & Bourke, 2011). Information relating to symptoms, examinations, and diagnoses in the database are recorded in a hierarchical clinical coding system called Read Codes (Booth, 1994). Prescription records, investigation results and lifestyle data are also captured within the database. IMRD-UK has been used extensively in epidemiological research and more recently used to examine mental health outcomes and prescription use (Chandan et al., 2019a, 2019b, 2020).

The database comprises of electronic medical records taken from 787 general practices throughout the UK (around 10% of all practices), which spread across four nations, comprised of a mix of deprived/non-deprived and urban/non-urban areas who utilise the Vision software system, meaning the number of contributing practices can vary over time. In order to ensure high quality data by avoiding biases relating to ‘immortal periods’, record updates and under-reporting, general practices were included 12 months following their installment of electronic practice records or from the practice's acceptable mortality recording date (Maguire, Blak, & Thompson, 2009). We utilised patient records taken from all of the eligible practices during the study period. Data extraction was facilitated using the data extraction for epidemiological research (DExtER) tool (Gokhale et al., 2021).

The purpose of this cohort study was to compare exposed patients (those with a Read code identifying confirmed use/misuse of cannabis) with unexposed patients (those without such codes) and then calculate their mental ill health outcomes defined through Read codes (composite measure; anxiety, depression and SMI) or the requirement of an incident prescription of medication used to treat mental ill health (composite measure; anti-depressants, anxiolytics and anti-psychotics).

### Exposure and outcome definition

Codes relating to GP recorded exposure to cannabis and mental ill health, which were dichotomous outcomes, were selected with the assistance of general practitioners and a public health clinician with a psychiatry background (Read code selection methodology previously reported; see Chandan et al., 2019a). GP recorded diagnoses of depression disorders and SMI (schizophrenia, psychosis, bipolar disorder) were expected to be well coded because they form part of the Quality Outcomes Framework (QOF; a payment incentivised performance indicator system), whereas anxiety disorders were anticipated to be well coded due to prevalence rates reported in IMRD-UK being very similar to self-reported national survey data (Martín-Merino, Ruigómez, Wallander, Johansson, & García-Rodríguez, 2010; McManus, Bebbington, Jenkins, & Brugha, 2016; NHS Digital, 2019). To act as an alternative form of validation for the diagnosis of mental ill health, we also evaluated prescriptions used to treat mental ill health as an outcome measure of interest. The prescription medication lists used to treat mental ill health were defined by use of the British National Formulary (BNF) chapters specific to each mental ill health condition (antidepressants, anxiolytics and antipsychotics; see the British National Formulary, 2019). Externalising disorders including risks for developing a substance use disorder, psychopathy or other personality disorders were not included as outcomes. Although we believe these outcomes would be of interest, their recorded validity in the dataset has not been previously explored, whereas the outcomes chosen for the study (depression, anxiety and SMI) have been present in the QOF during this study period so we would expect more accurate coding practice by GPs. In primary care data it was not possible to be truly certain of the temporality of coding as codes might appear if the GP chose to record them as a previous mental health diagnosis or as part of a new consultation, so we considered to code for incident only outcomes whereby we excluded those with the condition at baseline to provide a more accurate assessment of the risk of developing mental ill health following exposure to cannabis use.

The Read code list for cannabis use is provided on Supplementary Materials p2.

### Selection of unexposed group

Each exposed patient was matched with up to two unexposed control patients, who had no previously documented Read code relating to the exposure. Controls were matched by age, gender, smoking status, Townsend deprivation index and General Practice at index date. The same index date was assigned to controls to mitigate the potential of immortality time bias (Lévesque, Hanley, Kezouh, & Suissa, 2010).

### Follow-up period

The follow-up period for each patient was the time at risk of developing mental ill health from the index date until the exit date. The index date for those in the exposed group was the date of the first Read code relating to exposure during the study period (incident cases) or when they became eligible to enter the study for those with a previous history of exposure (prevalent cases). The exit date was defined as the earliest of the study end date, the last date of data collection from a given general practice, the date that the patient transferred from general practice, the date of death, or the date that the outcome of interest occurred.

### Covariates

The covariates that we adjusted for in our modelling were selected because of their independent association with the development of mental ill health (Sideli et al., 2020). These covariates were age, sex, Townsend deprivation score for the general practice (Townsend, Phillimore, & Beattie, 1988), body mass index (BMI), smoking and alcohol use which were recorded at baseline. The Townsend deprivation score is a measure of material deprivation within a locality, incorporating information on unemployment, household overcrowding, and car or home ownership; A higher score indicates greater socioeconomic deprivation (Townsend et al., 1988). Information was also reported on concurrent other illicit drug use (heroin, cocaine and amphetamine use) at baseline and a sensitivity analysis was carried out where those with additional drug use were excluded.

### Outcomes

We first calculated the risk of developing mental ill health (as separate measures of anxiety disorders, depression disorders, and SMI as defined through use of Read codes) and secondly of an incident prescription of medication used to treat mental ill health (measures comprising use of anxiolytics or antidepressants or antipsychotics) in exposed *versus* matched unexposed patients.

### Statistical analysis

Categorical baseline data were described as proportions and continuous data were described as means with standard deviations (SDs) or medians with inter-quartile ranges (IQRs). Missing baseline characteristic data were noted. Missing data in our covariates were treated as a separate missing category, and these data were included in the final analysis. To describe the presence of mental ill health at baseline, we used logistic regression to estimate unadjusted OR and adjusted OR after adjustment for key covariates (age, sex, Townsend deprivation score, BMI, smoking status and alcohol use).

To calculate an incidence rate (per 1000 person-years) for each of the outcomes of interest, patients with pre-existing illness (defined as a mental ill health code or prescription used to treat mental ill health on the index date) were excluded, to ensure the incidence rate reflected outcomes that occurred after cohort entry. We then used Cox regression offsetting for person-years of follow-up to calculate a hazard rate ratio (HRR) for each outcome of interest during the study period comparing those exposed with those not exposed. After adjustment for the same covariates, we calculated an adjusted HRR (aHRR). ORs and HRRs are presented with 95% confidence intervals (CIs) with statistical significance set at *p* < 0.05. We used Stata version 15.1 MP/4 software for all analyses.

### Sensitivity analysis

Two sensitivity analyses were conducted. The first was to examine if the findings differed when looking at incident only cases (those with an index date during the study period). The second was conducted to examine if the effects differed once all patients who had concurrent use of heroin, cocaine and amphetamines were excluded.

### Subgroup analysis

Two further subgroup analyses were conducted. We explored the impact of age and gender at GP recorded cannabis exposure date on the outcomes. Only incident patients (those who were both eligible for inclusion and were given a new recorded exposure of cannabis use during the study period) were included in the age subgroup analysis, whereas gender subgroup analysis was done in the whole cohort and incident only cohort separately. Patients were split into four categories, those with exposure <20, 20–24, 25–34 and 35+ years of age for the age subgroup analyses and into male and female for the gender subgroup analyses.

## Results

During the study period, there were 10 489 571 patients from 787 general practices who were eligible to participate in this study following application of practice and patient inclusion criteria. Of those patients, we identified 28 218 who had a recorded exposure to cannabis. These patients were matched to 56 208 unexposed control patients with no recorded exposure to cannabis, who met the matching criteria. Both the exposed and unexposed groups were followed up for almost 3 years. Median age at cohort entry (30 years), sex distribution (77% male), Townsend deprivation score and smoking status (76% current smokers) were very similar between the groups due to matching. Additionally, median BMI values and ethnicity ratios were similar between the groups, although there were high levels of missing ethnicity data.

Notably, the exposed group also had much higher rates of misusing drugs other than cannabis, this included heroin (5.9%), cocaine (4.6%) and amphetamines (2.6%) compared to significantly lower rates in the unexposed group (1.0, 0.3 and 0.2%).

Further details relating to the baseline characteristics of both groups can be seen in Table 1.

**Table 1.** Baseline sociodemographic and clinical characteristics of study participants

	Exposed (*n* = 28 218)	Unexposed (*n* = 56 208)	Exposed patients without a mental health diagnosis and prescription (*n* = 9212)	Exposed patients without a mental health diagnosis or prescription (*n* = 17 484)
Mean follow-up period (s.d.)	4.1 (3.8)	4.0 (3.6)	4.4 (3.9)	4.2 (3.8)
Median follow-up period (IQR)	2.9 (1.1–5.9)	2.9 (1.2–5.9)	3.3 (1.3–6.5)	3.0 (1.2–6.2)
Sex Males (%)	21 747 (77.1%)	43 314 (77.1%)	7904 (85.8%)	14 228 (81.4%)
Age in years (IQR)	30.0 (23.6–38.5)	30.1 (23.6–38.5)	25.9 (20.4–33.6)	28.1 (22.0–36.2)
Age categories in years (%)				
0–20	3242 (11.5%)	6277 (11.2%)	2140 (23.2%)	2884 (16.5%)
20–30	10 824 (38.4%)	21 708 (38.6%)	3830 (41.6%)	7093 (40.6%)
30–40	8071 (28.6%)	16 010 (28.5%)	2073 (22.5%)	4482 (25.6%)
>40	6081 (21.6%)	12 213 (21.7%)	1169 (12.7%)	3025 (17.3%)
Townsend (%)				
(Least deprived) 1	2170 (7.7%)	4237 (7.5%)	888 (9.6%)	1492 (8.5%)
2	2699 (9.6%)	5155 (9.2%)	1044 (11.3%)	1858 (10.6%)
3	4643 (16.5%)	8762 (15.6%)	1616 (17.5%)	2951 (16.9%)
4	6513 (23.1%)	12 034 (21.4%)	2029 (22.0%)	3939 (22.5%)
(Most deprived) 5	7844 (27.8%)	13 553 (24.1%)	2269 (24.6%)	4580 (26.2%)
Missing	4349 (15.4%)	12 467 (22.2%)	1366 (14.8%)	2664 (15.2%)
Ethnicity (%)				
White	15 298 (54.2%)	24 621 (43.8%)	4390 (47.7%)	8962 (51.3%)
Black	490 (1.7%)	713 (1.3%)	211 (2.3%)	331 (1.9%)
South Asian	279 (1.0%)	1340 (2.4%)	130 (1.4%)	199 (1.1%)
Mixed race	97 (0.3%)	647 (1.2%)	39 (0.4%)	64 (0.4%)
Others	254 (0.9%)	359 (0.6%)	105 (1.1%)	168 (1.0%)
Missing	11 800 (41.8%)	28 528 (50.8%)	4337 (47.1%)	7760 (44.4%)
BMI- kg/m^2^ (IQR)	23.4 (20.8–27.0)	23.5 (21.0–27.0)	23.0 (20.5–26.1)	23.1 (20.6–26.4)
BMI Categories- kg/m^2^ (%)				
Underweight (<18.5)	1404 (5.0%)	2174 (3.9%)	402 (4.4%)	841 (4.8%)
Normal weight (18.5–24.9)	10 959 (38.8%)	22 223 (39.5%)	3302 (35.8%)	6595 (37.7%)
Overweight (25–29.9)	4714 (16.7%)	9763 (17.4%)	1231 (13.4%)	2549 (14.6%)
Obese (30–34.9)	1721 (6.1%)	3424 (6.1%)	375 (4.1%)	856 (4.9%)
Obese (>35)	928 (3.3%)	1732 (3.1%)	166 (1.8%)	419 (2.4%)
Missing	8492 (30.1%)	16 892 (30.1%)	3736 (40.6%)	6224 (35.6%)
Smoker categories (%)				
Non smoker	2011 (7.1%)	4016 (7.1%)	875 (9.5%)	1448 (8.3%)
Not current smoker	2843 (10.1%)	5647 (10.0%)	914 (9.9%)	1781 (10.2%)
Current smoker	21 446 (76.0%)	42 751 (76.1%)	6346 (68.9%)	12 667 (72.4%)
Missing	1918 (6.8%)	3794 (6.7%)	1077 (11.7%)	1588 (9.1%)
Drinking status (%)				
Non-drinker	3873 (13.7%)	6096 (10.8%)	1094 (11.9%)	2235 (12.8%)
Not current drinker	1513 (5.4%)	1164 (2.1%)	228 (2.5%)	674 (3.9%)
Current drinker	15 654 (55.5%)	31 085 (55.3%)	4661 (50.6%)	9240 (52.8%)
Missing	7178 (25.4%)	17 863 (31.8%)	3229 (35.1%)	5335 (30.5%)
Medical conditions (%)				
All mental ill health	13 655 (48.4%)	11 045 (19.7%)	–	2921 (16.7%)
Depression	9690 (34.3%)	7958 (14.2%)	–	1760 (10.1%)
Anxiety	5032 (17.8%)	4336 (7.7%)	–	1057 (6.0%)
Serious mental illness	2982 (10.6%)	974 (1.7%)	–	557 (3.2%)
Number of prescriptions for drugs to treat above (%)				
Combined prescriptions	16 085 (57.0%)	18 385 (32.7%)	–	5351 (30.6%)
Anxiolytics	5541 (19.6%)	6220 (11.1%)	–	1698 (9.7%)
Antidepressants	13 420 (47.6%)	14 310 (25.5%)	–	4061 (23.2%)
Antipsychotics	5643 (20.0%)	5581 (9.9%)	–	1443 (8.3%)
Other drug abuse (%)				
Heroin	1653 (5.9%)	582 (1.0%)	336 (3.6%)	875 (5.0%)
Cocaine	1300 (4.6%)	190 (0.3%)	295 (3.2%)	703 (4.0%)
Amphetamine	736 (2.6%)	87 (0.2%)	135 (1.5%)	353 (2.0%)

It was evident at cohort entry that there was a greater burden of mental ill health in the exposed group than the unexposed group. In those exposed, 48.4% had a mental health diagnosis of any kind compared to 19.7% in the unexposed group. As seen in Table 1, depression was the most prevalent condition in both exposed (34.3%) and unexposed groups (14.2%), followed by anxiety (exposed: 17.8%; unexposed 7.7%) and SMI (exposed: 10.6%; unexposed 1.7%). A similar pattern was noted in the prescription of anti-depressants, anxiolytics and antipsychotics, with 57.0% of the exposed group having been prescribed mental health medication of any kind compared to 32.7% in the unexposed group. Following adjustment for confounders, this translated into an increased OR of mental ill health (all mental health diagnosis aOR 4.13; 95% CI 3.99–4.27, anxiety aOR 2.57; 95% CI 2.46–2.69, depression aOR 3.30; 95% CI 3.19–3.42 and SMI aOR 6.62; 95% CI 6.14–7.14) and prescription use of treatments for mental ill health (combined psychiatric prescription aOR 2.95; 95% CI 2.86–3.05, anxiolytics 1.97; 1.89–2.06, antidepressants 2.83; 2.74–2.92 and antipsychotics 2.24; 2.15–2.34, respectively). Further details can be seen in Table 1 and Table 2.

**Table 2.** ORs between unexposed and exposed populations in relation to mental illness outcomes and psychotropic drug prescriptions

	All mental ill health		Anxiety		Depression		Serious mental illness	
	Exposed	Unexposed	Exposed	Unexposed	Exposed	Unexposed	Exposed	Unexposed
Population	28 218	56 208	28 218	56 208	28 218	56 208	28 218	56 208
Number of patients with condition at baseline, n (%)	13 655 (48.39)	11 045 (19.65)	5032 (17.83)	4336 (7.71)	9690 (34.34)	7958 (14.16)	2982 (10.57)	974 (1.73)
Unadjusted OR (95% CI)	3.83 (3.72–3.96)		2.60 (2.49–2.71)		3.17 (3.06–3.28)		6.70 (6.22–7.21)	
*p* value	*p* < 0.01		*p* < 0.01		*p* < 0.01		*p* < 0.01	
Adjusted OR (95% CI)	4.13 (3.99–4.27)		2.57 (2.46–2.69)		3.30 (3.19–3.42)		6.62 (6.14–7.14)	
*p* value	*p* < 0.01		*p* < 0.01		*p* < 0.01		*p* < 0.01	
	Combined prescriptions		Anxiolytics		Antidepressants		Antipsychotics	
	Exposed	Unexposed	Exposed	Unexposed	Exposed	Unexposed	Exposed	Unexposed
Population	28 218	56 208	28 218	56 208	28 218	56 208	28 218	56 208
Number of patients with condition at baseline, n (%)	16 085 (57.00)	18 385 (32.71)	5541 (19.64)	6220 (11.07)	13 420 (47.56)	14 310 (25.46)	5643 (20.00)	5581 (9.93)
Unadjusted OR (95% CI)	2.73 (2.65–2.81)		1.96 (1.89–2.04)		2.66 (2.58–2.74)		2.27 (2.18–2.36)	
*p* value	*p* < 0.01		*p* < 0.01		*p* < 0.01		*p* < 0.01	
Adjusted OR (95% CI)	2.95 (2.86–3.05)		1.97 (1.89–2.06)		2.83 (2.74–2.92)		2.24 (2.15–2.34)	
*p* value	*p* < 0.01		*p* < 0.01		*p* < 0.01		*p* < 0.01	

Following exclusion of those with pre-existing mental ill health, during the study period, there were 1689, 1922 and 581 new diagnosis of anxiety disorders (IR 18.92/1000 py), depression (IR 27.69/1000 py) and SMI (IR 5.70/1000 py) in the exposed group compared to 1523, 2227 and 187 new diagnosis of anxiety (IR 7.55/1000 py), depression (IR 11.98/1000 py) and SMI (IR 0.85/1000 py) in the unexposed group. Following adjustment, this translated into an increased risk of developing anxiety (aHRR 2.46; 95% CI 2.29–2.64), depression (aHRR 2.34; 95% CI 2.20–2.49) and SMI (aHRR 6.41; 95% CI 5.42–7.57) in the exposed group when compared to the unexposed group. The exposed group also had significantly higher proportions of receiving medication for all three types of mental illnesses, however the difference between medications types was not as pronounced and the exposed group received a similarly increased risk of receiving all types of prescriptions (when compared to the unexposed group); antipsychotics (aHRR 2.13, 95% CI 1.99–2.28), anxiolytics (aHRR 1.84, 95% CI 1.73–1.95) and antidepressants (aHRR 2.28, 95% CI 2.18–2.38). Further details can be found in Table 3.

**Table 3.** Main study results in relation to mental illness outcomes and psychotropic drug prescriptions

	All mental ill health		Anxiety		Depression		Serious mental illness	
	Exposed	Unexposed	Exposed	Unexposed	Exposed	Unexposed	Exposed	Unexposed
Population	14 563	45 163	23 186	51 872	18 528	48 250	25 236	55 234
Outcome events, n (%)	2479 (17.02)	2964 (6.56)	1689 (7.28)	1523 (2.94)	1922 (10.37)	2227 (4.62)	581 (2.30)	187 (0.34)
Person-years	52 491	171 750	89 269	201 807	69 406	185 842	102 005	219 882
Crude incidence rate/1000 person years	47.23	17.26	18.92	7.55	27.69	11.98	5.70	0.85
Follow-up years, median (IQR)	2.48 (0.95–5.27)	2.76 (1.10–5.57)	2.72 (1.05–5.61)	2.86 (1.14–5.72)	2.63 (1.01–5.46)	2.81 (1.13–5.65)	2.90 (1.13–5.90)	2.93 (1.19–5.87)
Unadjusted HRR (95% CI)	2.73 (2.59–2.88)		2.51 (2.34–2.69)		2.31 (2.17–2.46)		6.76 (5.74–7.98)	
*p* value	*p* < 0.01		*p* < 0.01		*p* < 0.01		*p* < 0.01	
Adjusted HRR (95% CI)	2.73 (2.59–2.88)		2.46 (2.29–2.64)		2.34 (2.20–2.49)		6.41 (5.42–7.57)	
*p* value	*p* < 0.01		*p* < 0.01		*p* < 0.01		*p* < 0.01	
	Combined prescriptions		Anxiolytics		Antidepressants		Antipsychotics	
	Exposed	Unexposed	Exposed	Unexposed	Exposed	Unexposed	Exposed	Unexposed
Population	12 133	37 823	22 677	49 988	14 798	41 898	22 575	50 627
Outcome events, *n* (%)	3471 (28.61)	5519 (14.59)	1939 (8.55)	2428 (4.86)	3429 (23.17)	4766 (11.38)	1562 (6.92)	1676 (3.31)
Person-years	38 170	134 469	84 942	190 919	50 009	154 887	86 817	196 248
Crude incidence rate/1000 person years	90.94	41.04	22.83	12.72	68.57	30.77	17.99	8.54
Follow-up years, median (IQR)	2.04 (0.73–4.50)	2.51 (0.99–5.13)	2.57 (0.97–5.45)	2.77 (1.10–5.57)	2.22 (0.82–4.90)	2.63 (1.03–5.38)	2.65 (1.01–5.61)	2.83 (1.14–5.67)
Unadjusted HRR (95% CI)	2.19 (2.10–2.29)		1.80 (1.70–1.92)		2.21 (2.12–2.31)		2.13 (1.99–2.28)	
*p* value	*p* < 0.01		*p* < 0.01		*p* < 0.01		*p* < 0.01	
Adjusted HRR (95% CI)	2.27 (2.17–2.37)		1.84 (1.73–1.95)		2.28 (2.18–2.38)		2.13 (1.99–2.28)	
*p* value	*p* < 0.01		*p* < 0.01		*p* < 0.01		*p* < 0.01	

An initial sensitivity analysis was conducted to examine the incident only cases during the study period. There were 10 988 (39.6%) exposed incident patients who were matched to 21 894 unexposed patients. The baseline characteristics of this group were largely similar to the main cohort. At baseline there was similarly an increased OR of having anxiety (OR 2.35; 95% CI 2.18–2.53), depression (OR 2.90; 95% CI 2.73–3.09) and SMI (OR 4.24; 95% CI 3.71–4.84) in the exposed group compared to the unexposed group. The pattern was also similar for the OR describing the OR for prescription medication use at baseline. There was additionally an increased risk of going onto develop anxiety (aHRR 2.73; 95%CI 2.46–3.03), depression (aHRR 2.63; 95% CI 2.40–2.88) and SMI (aHRR 5.97; 95% CI 4.72–7.56) in the exposed group compared to the unexposed group. There was additionally an increased risk of requiring prescription treatments for mental ill health. Further details can be found on Supplementary Materials p3–6.

A second sensitivity analysis was carried out which excluded people who used heroin, cocaine or amphetamine at baseline for both the exposed and unexposed groups. The baseline results showed little change after exclusion. The baseline OR for having any type of mental ill health or need for prescription was similar to the main cohort analysis. The risk of developing mental ill health was also similar to the main cohort (anxiety aHRR 2.49; 95% CI 2.31–2.68, depression aHRR 2.36; 95% CI 2.21–2.52 and SMI aHRR 6.47; 95% CI 5.41–7.74). Further details can be found in Supplementary Materials p7–10.

During our subgroup analysis examining outcomes by age of exposure, we found similar adjusted HRRs across four age groups (<20, 20–24, 25–34, and 35+ years) when comparing common mental health disorders (depression and anxiety) between the exposed and the unexposed groups; however, the adjusted HRRs for SMI are particularly pronounced (although not statistically significantly different) in those with an exposure age of younger than 35 years (<20; aHRR 7.39; 95% CI 4.44–12.30: 20–24; 6.63; 4.16–10.57: 25–34; 7.02; 4.27–11.53: 35+; 4.08; 2.62–6.35 respectively) compared with those of the same age unexposed to cannabis. Further details can be found on Supplementary Materials p11–18. Another set of subgroup analyses were carried out by gender and again we found similar adjusted HRRs for males and females for both common mental health disorders and SMI. However, males were more likely to have been prescribed antipsychotic medications compared to females. Full details of the analyses can be found in Supplementary Materials (p19–p22).

## Discussion

To our knowledge, this is the first attempt to examine the relationship between cannabis use and development of mental ill health or future use of prescription medication (used to treat mental ill health) in the UK using a large representative primary care record dataset. Our study therefore provides an urgently needed addition to the current evidence base. We found that during a relatively short follow-up period, exposure to cannabis was associated with a four-fold risk of developing any mental disorder, a two-to-three-fold risk of developing anxiety, depression and a particularly substantial (almost seven-fold) risk of developing an SMI. The latter appears to be associated with a younger age of exposure. Use of cannabis was also related to a two-fold increase of being prescribed psychotropic medication for the treatment of mental illnesses. Even after accounting for misuse of other illicit substances, confounding and other factors which may influence the direction of causality of the relationship, these associations persisted. Nonetheless, this study was not a trial designed to detect causality or to draw causal conclusions.

Our results support the global literature which associates cannabis use with mental ill health. The ORs from our study were generally higher than those from previous meta-analyses when examining the relationship with affective disorders, which may be reflective of GP recordings of cannabis use relating to either higher levels of dependency or greater potency of cannabis use prior to reporting to the GP. In contrast to the known literature, recorded heroin use was much higher than results based on community surveys and the pattern was reversed for amphetamines (https://digital.nhs.uk/data-and-information/publications/statistical/statistics-on-drug-misuse/2020). This may be due to individuals only reporting addictions or serious substance misuse to their GP when it is a health problem and given the higher propensity for heroin to cause dependence than amphetamine, it is not surprising that the former poses a more serious health issue in individuals who may be misusing multiple drugs and for whom the consequences of such are more severe.

Interestingly in our study, exposure to cannabis was also substantially linked with the development of more common mental disorders such as depression and anxiety, with risks higher than previously inconsistent findings. These findings were still present in both sensitivity analyses, suggesting there is still a clear relationship between cannabis use and subsequent depression and anxiety, additionally confirmed by use of anxiolytics and anti-depressants. Despite these differences, it is clear in this study that there is a significant association with a substantial burden of mental ill health (both common and severe mental disorders) associated with cannabis use within the UK.

As a result, these findings are of crucial importance in the UK and beyond, given its novel contribution to the potential need for a public health approach to the management of people misusing cannabis and to the psychoeducation of the public regarding illicit drug use. The notion of cannabis being a safe drug may well be mistaken (Best, Gross, Manning, & Strang, 2005; Miller & Plant, 2002). Interestingly, the role cannabis plays in the wider mental health picture particularly in young people needs to be explored further in the UK, as regional rates of mental ill health are already on a steady increase in both adolescents and adults (Davis et al., 2019). It may be useful to further educate the wider public about the potential harm of misusing cannabis; we need to monitor closely the mental wellbeing those who are already frequent users. This is especially key in those with a genetic and/or social predisposition to developing mental illnesses.

Although this study elicited highly significant associations between cannabis use and mental ill health in one of the largest cohorts ever explored, there are still limitations with this approach. Ultimately, the quality of the data is inextricably linked to the accuracy of recoding by the clinician (in this case, primary care clinician) responsible for updating the electronic health records. Although there has been no formal validation on the use of Read codes in ascertaining cannabis use or mental health outcomes, we did select the read codes with the help of those trained in psychiatry. However, it is clear that there is a gross under-recording of cannabis use in GP records, as seen by the prevalence of recorded cannabis exposure substantially lower than self-reported survey records (Office of National Statistics, 2018). Similarly, accuracy in the recording of mental health outcomes in GP surgeries might also vary across regions or even practices. Barriers to reporting of substance misuse disorders are extensive in a primary care setting (McNeely et al., 2018), with factors both relating to the willingness of patients to declare their exposure as well as barriers to GPs enquiring about drug use. A low recorded prevalence is also likely to lead to a misclassification bias. This could lead to an underestimation of the effect size, or alternatively if primary care records only capture the most severe cases reflect an overestimation of risk. Although it is not possible from this study to identify why specific patients have been coded as exposed or unexposed, a recent qualitative study explored the rationale for GPs to record drug misuse in UK primary care electronic health records. It was found that the decision to record in the notes related to the GP's opinion on (1) their ongoing relationship with the patient, (2) the importance of patient choice in medical recording and (3) the need for transparent notes to aid future diagnoses and decision making (Davies-Kershaw, Petersen, Nazareth, & Stevenson, 2018). Although we do not anticipate this to create a systematic difference in the characteristics of patients misclassified due to the gross under-recording of cannabis exposure in this cohort, the generalisability of our study's findings must be interpreted cautiously.

Another limitation is that although we matched on the Townsend social deprivation score, it could be that the most socially disadvantaged individuals would simply never have registered with a GP or would have had unstable housing which would have required them to move frequently, leading to potential biases in sampling. Further, the Townsend social deprivation score in this dataset was an area-level indicator associated with the patient's postcode (Blak et al., 2011), which leads to intrinsic difficulties in accurately estimating the impact of sociodemographic and socioeconomic factors on the relationship between cannabis use and mental ill health. Given that this group of individuals have much higher rates of illicit drug use as well as untreated mental illness, our current study is unlikely to be representative of this most vulnerable population. Other factors often relevant in this population such as childhood maltreatment, trauma and their complex links with diagnoses other than depression, anxiety and SMI (e.g. personality disorders) and substance misuse are not explored in the current study. Reports of low mood and anxiety may also have been exacerbated by unstable social situations related to housing, employment and education. As approximately half of the ethnicity codes were missing, taking into account the strong associations between ethnic groups, deprivation and substance misuse, we have been unable to wholly account for the effect of this potential mediating factor on the relationship between substance misuse and mental ill health. We recommend further research is conducted in cohorts with more complete ethnicity data to confirm the robustness of these findings.

Additionally, due to the granularity of primary care recording of cannabis use, we were unable to the determine the type and potency of cannabis-containing products which led to exposed status, nor the frequency of use. Future studies should aim to sub-group by the type of cannabis-containing products used and record the frequency of use. There is an increased likelihood towards younger individuals developing SMI during their period of follow up, as it is usually less typical for an SMI to onset later in life and older adults with a developing SMI might not have been accurately captured by their GP records which could lead to an increased magnitude of the effect size in the younger age groups below 35 years. Lastly, although we were confident in recording of mental health diagnoses in the dataset, we had included a secondary outcome examining initiation of mental health medications. As it was not possible to exactly identify the dose given to the patients, it is important to note that some of the drugs included in our definition (e.g. amitriptyline) may be used to treat other conditions such as headache, neuropathic pain or sleep disorders. Some of the cannabis users may also have been misusing prescribed medications such as benzodiazepines. Despite these limitations, our findings remain notable as they suggest that any kind of recorded exposure to cannabis is moderately to severely associated with mental ill health outcomes.

## Conclusion

Our study found highly significant associations between cannabis use and increased risks of developing common and severe mental illnesses. Our findings have the potential to influence national policy on illicit drug use, which are especially pertinent due to the perceived ‘low risk’ status of cannabis by a proportion of the public, in policy statements and also reflecting how far cannabis is a focus of treatment provided by substance misuse services. In order to prevent outcomes of mental ill health in later life and decelerate the increasing trend in disease burden, primary care clinicians need to actively enquire about, monitor and discourage the use of cannabis in young people who may be particularly vulnerable.

## Supplementary material

For supplementary material accompanying this paper visit https://doi.org/10.1017/S003329172100386X. click here to view supplementary material
