# Supplementary material for: Associations between primary care recorded cannabis use and mental ill health in the UK: a population-based retrospective cohort study using UK primary care data
Source: Psychol Med. 2021 Oct 1;53(5):2106–15. doi: 10.1017/S003329172100386X (PMC10106282; doi:10.1017/S003329172100386X)
Supplement: Supplementary file 1 [file S003329172100386Xsup001.docx]

Supplementary Materials

Table of Contents

[Read codes relating to cannabis use 2](#_Toc79704885)

[Supplementary Table 1: Baseline characteristics of incident only cohort 3](#_Toc79704886)

[Supplementary Table 2: Odds ratio of incident only cohort 5](#_Toc79704887)

[Supplementary Table 3: Main Results of incident only cohort 6](#_Toc79704888)

[Supplementary Table 4: Baseline characteristics of excluding other drugs analysis 7](#_Toc79704889)

[Supplementary Table 5: Odds ratio of excluding other drugs analysis 9](#_Toc79704890)

[Supplementary Table 6: Main Results of excluding other drugs analysis 10](#_Toc79704891)

[Supplementary Table 7: (Age subgroup analysis of incident only cohort) Baseline characteristics 11](#_Toc79704892)

[Supplementary Table 8: (Age subgroup analysis of incident only cohort) Odds Ratio 13](#_Toc79704893)

[Supplementary Table 9: (Age subgroup analysis of incident only cohort) Main Results 16](#_Toc79704894)

***Supplementary Table 10: Gender subgroup analysis of whole cohort………………………………19***

***Supplementary Table 11: Gender subgroup analysis of incident only cohort……………………21***

[STROBE checklist 23](#_Toc79704895)

# Read codes relating to cannabis use

| Read code | Description |
| --- | --- |
| 13cE.00 | Prolonged high dose use of cannabis |
| 1T8..00 | H/O cannabis misuse |
| 1T80.00 | H/O daily cannabis misuse |
| 1T81.00 | H/O weekly cannabis misuse |
| 1T82.00 | H/O infrequent cannabis misuse |
| 1T83.00 | Previous history of cannabis misuse |
| E243.00 | Cannabis type drug dependence |
| E243000 | Cannabis dependence, unspecified |
| E243100 | Cannabis dependence, continuous |
| E243200 | Cannabis dependence, episodic |
| E243300 | Cannabis dependence in remission |
| E243z00 | Cannabis drug dependence NOS |
| E252.00 | Nondependent cannabis abuse |
| E252000 | Nondependent cannabis abuse, unspecified |
| E252100 | Nondependent cannabis abuse, continuous |
| E252200 | Nondependent cannabis abuse, episodic |
| E252300 | Nondependent cannabis abuse in remission |
| E252z00 | Nondependent cannabis abuse NOS |
| Eu12211 | [X]Drug addiction - cannabis |

# Supplementary table 1: Baseline characteristics of incident only cohort

|  | **Exposed (n=10,988)** | **Unexposed (n=21,894)** |
| --- | --- | --- |
| **Mean follow-up period (SD)** | 4.8 (3.9) | 4.1 (3.6) |
| **Median follow-up period (IQR)** | 3.8 (1.6-7.1) | 3.0 (1.2-6.1) |
| **Sex- Males (%)** | 8,688 (79.1%) | 17,318 (79.1%) |
| **Age in years (IQR)** | 27.3 (21.0-37.4) | 27.3 (21.0-37.4) |
| **Age Categories in years (%)** |  |  |
| 0-20 | 2,233 (20.3%) | 4,313 (19.7%) |
| 20-30 | 4,182 (38.1%) | 8,412 (38.4%) |
| 30-40 | 2,373 (21.6%) | 4,731 (21.6%) |
| >40 | 2,200 (20.0%) | 4,438 (20.3%) |
| **Townsend (%)** |  |  |
| (Least deprived) 1 | 953 (8.7%) | 1,834 (8.4%) |
| 2 | 1,049 (9.5%) | 1,998 (9.1%) |
| 3 | 1,790 (16.3%) | 3,365 (15.4%) |
| 4 | 2,471 (22.5%) | 4,529 (20.7%) |
| (Most deprived) 5 | 3,189 (29.0%) | 5,440 (24.8%) |
| Missing | 1,536 (14.0%) | 4,728 (21.6%) |
| **Ethnicity (%)** |  |  |
| White | 4,625 (42.1%) | 8,937 (40.8%) |
| Black | 160 (1.5%) | 252 (1.2%) |
| South Asian | 115 (1.0%) | 450 (2.1%) |
| Mixed Race | 28 (0.3%) | 229 (1.0%) |
| Others | 71 (0.6%) | 144 (0.7%) |
| Missing | 5,989 (54.5%) | 11,882 (54.3%) |
| **BMI- kg/m2 (IQR)** | 22.9 (20.4-26.7) | 23.1 (20.6-26.6) |
| **BMI Categories- kg/m2 (%)** |  |  |
| Underweight (<18.5) | 638 (5.8%) | 966 (4.4%) |
| Normal weight (18.5-24.9) | 3,861 (35.1%) | 7,938 (36.3%) |
| Overweight (25-29.9) | 1,512 (13.8%) | 3,128 (14.3%) |
| Obese(30-34.9) | 542 (4.9%) | 1,072 (4.9%) |
| Obese(>35) | 324 (2.9%) | 600 (2.7%) |
| Missing | 4,111 (37.4%) | 8,190 (37.4%) |
| **Smoker Categories (%)** |  |  |
| Non Smoker | 948 (8.6%) | 1,893 (8.6%) |
| Not Current Smoker | 839 (7.6%) | 1,662 (7.6%) |
| Current Smoker | 8,350 (76.0%) | 16,645 (76.0%) |
| Missing | 851 (7.7%) | 1,694 (7.7%) |
| **Drinking Status (%)** |  |  |
| Non Drinker | 1,309 (11.9%) | 2,285 (10.4%) |
| Not Current Drinker | 535 (4.9%) | 428 (2.0%) |
| Current Drinker | 5,634 (51.3%) | 10,831 (49.5%) |
| Missing | 3,510 (31.9%) | 8,350 (38.1%) |

| **Medical Conditions (%)** | | |
| --- | --- | --- |
| All Mental Ill Health | 4,411 (40.1%) | 3,986 (18.2%) |
| Depression | 3,215 (29.3%) | 2,845 (13.0%) |
| Anxiety | 1,707 (15.5%) | 1,569 (7.2%) |
| Serious Mental Illness | 724 (6.6%) | 349 (1.6%) |
| **Number of Prescriptions for drugs to treat above (%)** | | |
| Combined Prescriptions | 6,382 (58.1%) | 6,662 (30.4%) |
| Anxiolytics | 2,393 (21.8%) | 2,267 (10.4%) |
| Antidepressants | 5,372 (48.9%) | 5,132 (23.4%) |
| Antipsychotics | 2,302 (21.0%) | 2,092 (9.6%) |
| **Other drug abuse (%)** | | |
| Heroin | 335 (3.0%) | 197 (0.9%) |
| Cocaine | 327 (3.0%) | 60 (0.3%) |
| Amphetamine | 132 (1.2%) | 28 (0.1%) |

|  | **All Mental Ill Health** | | **Anxiety** | | **Depression** | | **Serious Mental Illness** | |
| --- | --- | --- | --- | --- | --- | --- | --- | --- |
|  | **Exposed** | **Unexposed** | **Exposed** | **Unexposed** | **Exposed** | **Unexposed** | **Exposed** | **Unexposed** |
| Population | 10,988 | 21,894 | 10,988 | 21,894 | 10,988 | 21,894 | 10,988 | 21,894 |
| Number of patients with condition at baseline, n (%) | 4,411 (40.14) | 3,986 (18.21) | 1,707 (15.54) | 1,569 (7.17) | 3,215 (29.26) | 2,845 (12.99) | 724 (6.59) | 349 (1.59) |
| Unadjusted Odds Ratio (95% Confidence interval) | 3.01 (2.86-3.17) | | 2.38 (2.22-2.56) | | 2.77 (2.62-2.93) | | 4.35 (3.82-4.96) | |
| p-value | p<0.01 | | p<0.01 | | p<0.01 | | p<0.01 | |
| Adjusted Odds Ratio (95% CI) | 3.25 (3.08-3.44) | | 2.35 (2.18-2.53) | | 2.90 (2.73-3.09) | | 4.24 (3.71-4.84) | |
| p-value | p<0.01 | | p<0.01 | | p<0.01 | | p<0.01 | |

# Supplementary Table 2: Odds ratio of incident only cohort

|  | **Combined Prescriptions** | | **Anxiolytics** | | **Antidepressants** | | **Antipsychotics** | |
| --- | --- | --- | --- | --- | --- | --- | --- | --- |
|  | **Exposed** | **Unexposed** | **Exposed** | **Unexposed** | **Exposed** | **Unexposed** | **Exposed** | **Unexposed** |
| Population | 10,988 | 21,894 | 10,988 | 21,894 | 10,988 | 21,894 | 10,988 | 21,894 |
| Number of patients with condition at baseline, n (%) | 6,382 (58.08) | 6,662 (30.43) | 2,393 (21.78) | 2,267 (10.35) | 5,372 (48.89) | 5,132 (23.44) | 2,302 (20.95) | 2,092 (9.56) |
| Unadjusted Odds Ratio (95% Confidence interval) | 3.17 (3.02-3.32) | | 2.41 (2.26-2.57) | | 3.12 (2.98-3.28) | | 2.51 (2.35-2.68) | |
| p-value | p<0.01 | | p<0.01 | | p<0.01 | | p<0.01 | |
| Adjusted Odds Ratio (95% CI) | 3.76 (3.56-3.96) | | 2.55 (2.38-2.73) | | 3.61 (3.42-3.81) | | 2.56 (2.39-2.74) | |
| p-value | p<0.01 | | p<0.01 | | p<0.01 | | p<0.01 | |

# Supplementary Table 3: Main Results of incident only cohort

|  | **All Mental Ill Health** | | **Anxiety** | | **Depression** | | **Serious Mental Illness** | |
| --- | --- | --- | --- | --- | --- | --- | --- | --- |
|  | **Exposed** | **Unexposed** | **Exposed** | **Unexposed** | **Exposed** | **Unexposed** | **Exposed** | **Unexposed** |
| Population | 6,577 | 17,908 | 9,281 | 20,325 | 7,773 | 19,049 | 10,264 | 21,545 |
| Outcome events, n (%) | 1,355 (20.60) | 1,227 (6.85) | 856 (9.22) | 617 (3.04) | 1,028 (13.23) | 919 (4.82) | 300 (2.92) | 92 (0.43) |
| Person-years | 25,988 | 70,248 | 40,756 | 81,891 | 32,717 | 75,593 | 47,723 | 88,627 |
| Crude Incidence Rate/1000 person years | 52.14 | 17.47 | 21.00 | 7.53 | 31.42 | 12.16 | 6.29 | 1.04 |
| Follow-up years, Median (IQR) | 2.90 (1.13-5.79) | 2.83 (1.14-5.72) | 3.38 (1.39-6.49) | 2.93 (1.17-5.95) | 3.20 (1.27-6.16) | 2.88 (1.16-5.81) | 3.65 (1.51-6.92) | 3.00 (1.22-6.07) |
| Unadjusted Hazard Rate ratio (95% CI) | 2.99 (2.77-3.23) | | 2.81 (2.53-3.11) | | 2.60 (2.38-2.84) | | 6.27 (4.97-7.92) | |
| p-value | p<0.01 | | p<0.01 | | p<0.01 | | p<0.01 | |
| Adjusted Hazard Rate ratio (95% CI) | 2.99 (2.76-3.23) | | 2.73 (2.46-3.03) | | 2.63 (2.40-2.88) | | 5.97 (4.72-7.56) | |
| p-value | p<0.01 | | p<0.01 | | p<0.01 | | p<0.01 | |

|  | **Combined Prescriptions** | | **Anxiolytics** | | **Antidepressants** | | **Antipsychotics** | |
| --- | --- | --- | --- | --- | --- | --- | --- | --- |
|  | **Exposed** | **Unexposed** | **Exposed** | **Unexposed** | **Exposed** | **Unexposed** | **Exposed** | **Unexposed** |
| Population | 4,606 | 15,232 | 8,595 | 19,627 | 5,616 | 16,762 | 8,686 | 19,802 |
| Outcome events, n (%) | 1,492 (32.39) | 2,256 (14.81) | 766 (8.91) | 954 (4.86) | 1,492 (26.57) | 1,966 (11.73) | 693 (7.98) | 649 (3.28) |
| Person-years | 16,667 | 56,047 | 37,868 | 77,622 | 21,845 | 64,003 | 39,055 | 79,509 |
| Crude Incidence Rate/1000 person years | 89.52 | 40.25 | 20.23 | 12.29 | 68.30 | 30.72 | 17.74 | 8.16 |
| Follow-up years, Median (IQR) | 2.56 (0.89-5.33) | 2.60 (1.03-5.31) | 3.36 (1.33-6.54) | 2.87 (1.14-5.78) | 2.80 (1.01-5.73) | 2.71 (1.08-5.54) | 3.43 (1.38-6.71) | 2.92 (1.17-5.91) |
| Unadjusted Hazard Rate ratio (95% CI) | 2.23 (2.09-2.38) | | 1.68 (1.53-1.85) | | 2.24 (2.09-2.39) | | 2.25 (2.02-2.51) | |
| p-value | p<0.01 | | p<0.01 | | p<0.01 | | p<0.01 | |
| Adjusted Hazard Rate ratio (95% CI) | 2.34 (2.18-2.50) | | 1.72 (1.56-1.89) | | 2.33 (2.17-2.49) | | 2.27 (2.03-2.53) | |
| p-value | p<0.01 | | p<0.01 | | p<0.01 | | p<0.01 | |

# Supplementary table 4: Baseline characteristics of excluding other drugs analysis

|  | **Exposed (n=25,078)** | **Unexposed (n=49,270)** |
| --- | --- | --- |
| **Mean follow-up period (SD)** | 4.2 (3.8) | 4.0 (3.6) |
| **Median follow-up period (IQR)** | 3.0 (1.2-6.1) | 3.0 (1.2-5.9) |
| **Sex- Males (%)** | 19,389 (77.3%) | 38,024 (77.2%) |
| **Age in years (IQR)** | 29.5 (23.2-38.2) | 29.4 (23.1-38.2) |
| **Age Categories in years (%)** |  |  |
| 0-20 | 3,149 (12.6%) | 6,089 (12.4%) |
| 20-30 | 9,883 (39.4%) | 19,642 (39.9%) |
| 30-40 | 6,760 (27.0%) | 13,119 (26.6%) |
| >40 | 5,286 (21.1%) | 10,420 (21.1%) |
| **Townsend (%)** |  |  |
| (Least deprived) 1 | 2,013 (8.0%) | 3,875 (7.9%) |
| 2 | 2,453 (9.8%) | 4,635 (9.4%) |
| 3 | 4,187 (16.7%) | 7,789 (15.8%) |
| 4 | 5,743 (22.9%) | 10,459 (21.2%) |
| (Most deprived) 5 | 6,854 (27.3%) | 11,547 (23.4%) |
| Missing | 3,828 (15.3%) | 10,965 (22.3%) |
| **Ethnicity (%)** |  |  |
| White | 13,283 (53.0%) | 21,283 (43.2%) |
| Black | 455 (1.8%) | 629 (1.3%) |
| South Asian | 253 (1.0%) | 1,150 (2.3%) |
| Mixed Race | 88 (0.4%) | 577 (1.2%) |
| Others | 242 (1.0%) | 313 (0.6%) |
| Missing | 10,757 (42.9%) | 25,318 (51.4%) |
| **BMI- kg/m2 (IQR)** | 23.4 (20.7-27.0) | 23.5 (21.0-27.0) |
| **BMI Categories- kg/m2 (%)** |  |  |
| Underweight (<18.5) | 1,244 (5.0%) | 1,890 (3.8%) |
| Normal weight (18.5-24.9) | 9,684 (38.6%) | 19,259 (39.1%) |
| Overweight (25-29.9) | 4,151 (16.6%) | 8,485 (17.2%) |
| Obese(30-34.9) | 1,516 (6.0%) | 2,996 (6.1%) |
| Obese(>35) | 805 (3.2%) | 1,510 (3.1%) |
| Missing | 7,678 (30.6%) | 15,130 (30.7%) |
| **Smoker Categories (%)** |  |  |
| Non Smoker | 1,889 (7.5%) | 3,772 (7.7%) |
| Not Current Smoker | 2,585 (10.3%) | 5,119 (10.4%) |
| Current Smoker | 18,845 (75.1%) | 36,905 (74.9%) |
| Missing | 1,759 (7.0%) | 3,474 (7.1%) |
| **Drinking Status (%)** |  |  |
| Non Drinker | 3,380 (13.5%) | 5,267 (10.7%) |
| Not Current Drinker | 1,254 (5.0%) | 956 (1.9%) |
| Current Drinker | 13,870 (55.3%) | 26,980 (54.8%) |
| Missing | 6,574 (26.2%) | 16,067 (32.6%) |

| **Medical Conditions (%)** | | |
| --- | --- | --- |
| All Mental Ill Health | 11,821 (47.1%) | 9,268 (18.8%) |
| Depression | 8,367 (33.4%) | 6,658 (13.5%) |
| Anxiety | 4,341 (17.3%) | 3,651 (7.4%) |
| Serious Mental Illness | 2,536 (10.1%) | 782 (1.6%) |
| **Number of Prescriptions for drugs to treat above (%)** | | |
| Combined Prescriptions | 13,950 (55.6%) | 15,540 (31.5%) |
| Anxiolytics | 4,648 (18.5%) | 5,136 (10.4%) |
| Antidepressants | 11,634 (46.4%) | 12,002 (24.4%) |
| Antipsychotics | 4,878 (19.5%) | 4,752 (9.6%) |

# Supplementary Table 5: Odds ratio of excluding other drugs analysis

|  | **All Mental Ill Health** | | **Anxiety** | | **Depression** | | **Serious Mental Illness** | |
| --- | --- | --- | --- | --- | --- | --- | --- | --- |
|  | **Exposed** | **Unexposed** | **Exposed** | **Unexposed** | **Exposed** | **Unexposed** | **Exposed** | **Unexposed** |
| Population | 25,078 | 49,270 | 25,078 | 49,270 | 25,078 | 49,270 | 25,078 | 49,270 |
| Number of patients with condition at baseline, n (%) | 11,821 (47.14) | 9,268 (18.81) | 4,341 (17.31) | 3,651 (7.41) | 8,367 (33.36) | 6,658 (13.51) | 2,536 (10.11) | 782 (1.59) |
| Unadjusted Odds Ratio (95% Confidence interval) | 3.85 (3.72-3.98) | | 2.62 (2.50-2.74) | | 3.20 (3.09-3.32) | | 6.98 (6.43-7.57) | |
| p-value | p<0.01 | | p<0.01 | | p<0.01 | | p<0.01 | |
| Adjusted Odds Ratio (95% CI) | 4.15 (4.00-4.30) | | 2.59 (2.47-2.72) | | 3.35 (3.22-3.48) | | 6.88 (6.33-7.47) | |
| p-value | p<0.01 | | p<0.01 | | p<0.01 | | p<0.01 | |

|  | **Combined Prescriptions** | | **Anxiolytics** | | **Antidepressants** | | **Antipsychotics** | |
| --- | --- | --- | --- | --- | --- | --- | --- | --- |
|  | **Exposed** | **Unexposed** | **Exposed** | **Unexposed** | **Exposed** | **Unexposed** | **Exposed** | **Unexposed** |
| Population | 25,078 | 49,270 | 25,078 | 49,270 | 25,078 | 49,270 | 25,078 | 49,270 |
| Number of patients with condition at baseline, n (%) | 13,950 (55.63) | 15,540 (31.54) | 4,648 (18.53) | 5,136 (10.42) | 11,634 (46.39) | 12,002 (24.36) | 4,878 (19.45) | 4,752 (9.64) |
| Unadjusted Odds Ratio (95% Confidence interval) | 2.72 (2.64-2.81) | | 1.95 (1.87-2.04) | | 2.69 (2.60-2.78) | | 2.26 (2.17-2.36) | |
| p-value | p<0.01 | | p<0.01 | | p<0.01 | | p<0.01 | |
| Adjusted Odds Ratio (95% CI) | 2.94 (2.85-3.05) | | 1.96 (1.87-2.05) | | 2.86 (2.77-2.97) | | 2.24 (2.14-2.34) | |
| p-value | p<0.01 | | p<0.01 | | p<0.01 | | p<0.01 | |

|  | **All Mental Ill Health** | | **Anxiety** | | **Depression** | | **Serious Mental Illness** | |
| --- | --- | --- | --- | --- | --- | --- | --- | --- |
|  | **Exposed** | **Unexposed** | **Exposed** | **Unexposed** | **Exposed** | **Unexposed** | **Exposed** | **Unexposed** |
| Population | 13,257 | 40,002 | 20,737 | 45,619 | 16,711 | 42,612 | 22,542 | 48,488 |
| Outcome events, n (%) | 2,259 (17.04) | 2,611 (6.53) | 1,520 (7.33) | 1,319 (2.89) | 1,747 (10.45) | 1,958 (4.59) | 515 (2.28) | 160 (0.33) |
| Person-years | 48,579 | 153,761 | 81,563 | 179,584 | 63,735 | 166,055 | 93,023 | 195,334 |
| Crude Incidence Rate/1000 person years | 46.50 | 16.98 | 18.64 | 7.34 | 27.41 | 11.79 | 5.54 | 0.82 |
| Follow-up years, Median (IQR) | 2.52 (0.98-5.36) | 2.80 (1.11-5.63) | 2.82 (1.09-5.74) | 2.90 (1.16-5.80) | 2.69 (1.03-5.55) | 2.85 (1.14-5.73) | 2.97 (1.16-6.01) | 2.98 (1.20-5.94) |
| Unadjusted Hazard Rate ratio (95% CI) | 2.73 (2.58-2.89) | | 2.54 (2.36-2.73) | | 2.33 (2.18-2.48) | | 6.84 (5.73-8.17) | |
| p-value | p<0.01 | | p<0.01 | | p<0.01 | | p<0.01 | |
| Adjusted Hazard Rate ratio (95% CI) | 2.73 (2.58-2.89) | | 2.49 (2.31-2.68) | | 2.36 (2.21-2.52) | | 6.47 (5.41-7.74) | |
| p-value | p<0.01 | | p<0.01 | | p<0.01 | | p<0.01 | |

# Supplementary Table 6: Main Results of excluding other drugs analysis

|  | **Combined Prescriptions** | | **Anxiolytics** | | **Antidepressants** | | **Antipsychotics** | |
| --- | --- | --- | --- | --- | --- | --- | --- | --- |
|  | **Exposed** | **Unexposed** | **Exposed** | **Unexposed** | **Exposed** | **Unexposed** | **Exposed** | **Unexposed** |
| Population | 11,128 | 33,730 | 20,430 | 44,134 | 13,444 | 37,268 | 20,200 | 44,518 |
| Outcome events, n (%) | 3,184 (28.61) | 4,908 (14.55) | 1,737 (8.50) | 2,113 (4.79) | 3,100 (23.06) | 4,242 (11.38) | 1,396 (6.91) | 1,477 (3.32) |
| Person-years | 35,720 | 121,132 | 78,247 | 170,538 | 46,307 | 139,249 | 79,355 | 174,510 |
| Crude Incidence Rate/1000 person years | 89.14 | 40.52 | 22.20 | 12.39 | 66.94 | 30.46 | 17.59 | 8.46 |
| Follow-up years, Median (IQR) | 2.12 (0.76-4.58) | 2.54 (1.00-5.21) | 2.66 (1.01-5.56) | 2.82 (1.12-5.64) | 2.29 (0.85-5.01) | 2.66 (1.04-5.45) | 2.74 (1.04-5.72) | 2.87 (1.15-5.75) |
| Unadjusted Hazard Rate ratio (95% CI) | 2.18 (2.08-2.28) | | 1.80 (1.69-1.92) | | 2.18 (2.08-2.29) | | 2.11 (1.96-2.27) | |
| p-value | p<0.01 | | p<0.01 | | p<0.01 | | p<0.01 | |
| Adjusted Hazard Rate ratio (95% CI) | 2.25 (2.15-2.36) | | 1.85 (1.73-1.97) | | 2.25 (2.14-2.36) | | 2.12 (1.97-2.28) | |
| p-value | p<0.01 | | p<0.01 | | p<0.01 | | p<0.01 | |

# Supplementary Table 7: (Age subgroup analysis of incident only cohort) Baseline characteristics

|  | **<20** | | **20-24** | | **25-34** | | **35<** | |
| --- | --- | --- | --- | --- | --- | --- | --- | --- |
|  | **Exposed (n=2,233)** | **Unexposed (n=4,437)** | **Exposed (n=2,409)** | **Unexposed (n=4,801)** | **Exposed (n=3,071)** | **Unexposed (n=6,117)** | **Exposed (n=3,275)** | **Unexposed (n=6,539)** |
| **Mean follow-up period (SD)** | 4.9 (3.9) | 4.2 (3.7) | 4.8 (3.8) | 3.9 (3.6) | 4.9 (4.0) | 4.1 (3.6) | 4.5 (3.8) | 4.2 (3.7) |
| **Median follow-up period (IQR)** | 3.9 (1.8-7.4) | 3.2 (1.2-6.3) | 3.9 (1.7-7.1) | 2.9 (1.1-5.7) | 3.9 (1.7-7.4) | 2.9 (1.2-6.0) | 3.4 (1.4-6.6) | 3.2 (1.3-6.2) |
| **Sex- Males (%)** | 1,761 (78.9%) | 3,502 (78.9%) | 1,943 (80.7%) | 3,873 (80.7%) | 2,482 (80.8%) | 4,948 (80.9%) | 2,502 (76.4%) | 4,995 (76.4%) |
| **Age in years (IQR)** | 17.8 (16.4-19.0) | 17.9 (16.5-19.1) | 22.3 (21.2-23.6) | 22.4 (21.2-23.6) | 29.3 (27.0-31.8) | 29.3 (27.1-31.8) | 43.2 (38.8-49.4) | 43.2 (38.8-49.4) |
| **Townsend (%)** | | | | | | | | |
| (Least deprived) 1 | 296 (13.3%) | 563 (12.7%) | 251 (10.4%) | 479 (10.0%) | 228 (7.4%) | 436 (7.1%) | 178 (5.4%) | 356 (5.4%) |
| 2 | 286 (12.8%) | 545 (12.3%) | 233 (9.7%) | 444 (9.2%) | 286 (9.3%) | 534 (8.7%) | 244 (7.5%) | 475 (7.3%) |
| 3 | 393 (17.6%) | 720 (16.2%) | 398 (16.5%) | 748 (15.6%) | 534 (17.4%) | 1,009 (16.5%) | 465 (14.2%) | 888 (13.6%) |
| 4 | 459 (20.6%) | 832 (18.8%) | 557 (23.1%) | 1,046 (21.8%) | 702 (22.9%) | 1,306 (21.4%) | 753 (23.0%) | 1,345 (20.6%) |
| (Most deprived) 5 | 522 (23.4%) | 879 (19.8%) | 604 (25.1%) | 1,092 (22.7%) | 909 (29.6%) | 1,584 (25.9%) | 1,154 (35.2%) | 1,885 (28.8%) |
| Missing | 277 (12.4%) | 898 (20.2%) | 366 (15.2%) | 992 (20.7%) | 412 (13.4%) | 1,248 (20.4%) | 481 (14.7%) | 1,590 (24.3%) |
| **Ethnicity (%)** | | | | | | | | |
| White | 769 (34.4%) | 1,444 (32.5%) | 932 (38.7%) | 1,781 (37.1%) | 1,308 (42.6%) | 2,594 (42.4%) | 1,616 (49.3%) | 3,118 (47.7%) |
| Black | 26 (1.2%) | 51 (1.1%) | 34 (1.4%) | 57 (1.2%) | 31 (1.0%) | 67 (1.1%) | 69 (2.1%) | 77 (1.2%) |
| South Asian | 16 (0.7%) | 60 (1.4%) | 32 (1.3%) | 105 (2.2%) | 41 (1.3%) | 159 (2.6%) | 26 (0.8%) | 126 (1.9%) |
| Mixed Race | (n<5) | 28 (0.6%) | 5 (0.2%) | 60 (1.2%) | 12 (0.4%) | 76 (1.2%) | 8 (0.2%) | 65 (1.0%) |
| Others | 14 (0.6%) | 35 (0.8%) | 16 (0.7%) | 34 (0.7%) | 20 (0.7%) | 43 (0.7%) | 21 (0.6%) | 32 (0.5%) |
| Missing | 1,405 (62.9%) | 2,819 (63.5%) | 1,390 (57.7%) | 2,764 (57.6%) | 1,659 (54.0%) | 3,178 (52.0%) | 1,535 (46.9%) | 3,121 (47.7%) |

| **BMI- kg/m2 (IQR)** | 21.0 (18.9-24.0) | 20.9 (19.0-23.9) | 21.7 (19.4-25.0) | 21.7 (19.7-25.1) | 22.7 (20.4-26.0) | 22.8 (20.6-25.9) | 24.3 (21.5-27.9) | 24.4 (21.7-28.0) |
| --- | --- | --- | --- | --- | --- | --- | --- | --- |
| **BMI Categories- kg/m2 (%)** | | | | | | | | |
| Underweight (<18.5) | 138 (6.2%) | 237 (5.3%) | 172 (7.1%) | 263 (5.5%) | 182 (5.9%) | 257 (4.2%) | 146 (4.5%) | 209 (3.2%) |
| Normal weight (18.5-24.9) | 383 (17.2%) | 799 (18.0%) | 763 (31.7%) | 1,589 (33.1%) | 1,320 (43.0%) | 2,735 (44.7%) | 1,395 (42.6%) | 2,815 (43.0%) |
| Overweight (25-29.9) | 78 (3.5%) | 155 (3.5%) | 197 (8.2%) | 413 (8.6%) | 442 (14.4%) | 911 (14.9%) | 795 (24.3%) | 1,649 (25.2%) |
| Obese(30-34.9) | 29 (1.3%) | 63 (1.4%) | 72 (3.0%) | 137 (2.9%) | 144 (4.7%) | 269 (4.4%) | 297 (9.1%) | 603 (9.2%) |
| Obese(>35) | 22 (1.0%) | 28 (0.6%) | 51 (2.1%) | 96 (2.0%) | 90 (2.9%) | 172 (2.8%) | 161 (4.9%) | 304 (4.6%) |
| Missing | 1,583 (70.9%) | 3,155 (71.1%) | 1,154 (47.9%) | 2,303 (48.0%) | 893 (29.1%) | 1,773 (29.0%) | 481 (14.7%) | 959 (14.7%) |
| **Smoker Categories (%)** | | | | | | | | |
| Non Smoker | 384 (17.2%) | 767 (17.3%) | 257 (10.7%) | 514 (10.7%) | 187 (6.1%) | 374 (6.1%) | 120 (3.7%) | 238 (3.6%) |
| Not Current Smoker | 73 (3.3%) | 136 (3.1%) | 159 (6.6%) | 315 (6.6%) | 240 (7.8%) | 479 (7.8%) | 367 (11.2%) | 732 (11.2%) |
| Current Smoker | 1,263 (56.6%) | 2,511 (56.6%) | 1,808 (75.1%) | 3,605 (75.1%) | 2,557 (83.3%) | 5,091 (83.2%) | 2,722 (83.1%) | 5,438 (83.2%) |
| Missing | 513 (23.0%) | 1,023 (23.1%) | 185 (7.7%) | 367 (7.6%) | 87 (2.8%) | 173 (2.8%) | 66 (2.0%) | 131 (2.0%) |
| **Drinking Status (%)** | | | | | | | | |
| Non Drinker | 234 (10.5%) | 344 (7.8%) | 256 (10.6%) | 487 (10.1%) | 358 (11.7%) | 667 (10.9%) | 461 (14.1%) | 787 (12.0%) |
| Not Current Drinker | 23 (1.0%) | 13 (0.3%) | 60 (2.5%) | 37 (0.8%) | 138 (4.5%) | 110 (1.8%) | 314 (9.6%) | 268 (4.1%) |
| Current Drinker | 518 (23.2%) | 691 (15.6%) | 1,097 (45.5%) | 1,927 (40.1%) | 1,855 (60.4%) | 3,661 (59.8%) | 2,164 (66.1%) | 4,552 (69.6%) |
| Missing | 1,458 (65.3%) | 3,389 (76.4%) | 996 (41.3%) | 2,350 (48.9%) | 720 (23.4%) | 1,679 (27.4%) | 336 (10.3%) | 932 (14.3%) |
| **Medical Conditions (%)** | | | | | | | | |
| All Mental Ill Health | 331 (14.8%) | 210 (4.7%) | 828 (34.4%) | 548 (11.4%) | 1,411 (45.9%) | 1,231 (20.1%) | 1,841 (56.2%) | 1,997 (30.5%) |
| Depression | 204 (9.1%) | 122 (2.7%) | 568 (23.6%) | 386 (8.0%) | 1,037 (33.8%) | 871 (14.2%) | 1,406 (42.9%) | 1,466 (22.4%) |
| Anxiety | 113 (5.1%) | 95 (2.1%) | 291 (12.1%) | 185 (3.9%) | 525 (17.1%) | 466 (7.6%) | 778 (23.8%) | 823 (12.6%) |
| Serious Mental Illness | 43 (1.9%) | 7 (0.2%) | 116 (4.8%) | 39 (0.8%) | 200 (6.5%) | 95 (1.6%) | 365 (11.1%) | 208 (3.2%) |
| **Number of Prescriptions for drugs to treat above (%)** | | | | | | | | |
| Combined Prescriptions | 566 (25.3%) | 444 (10.0%) | 1,281 (53.2%) | 1,051 (21.9%) | 2,030 (66.1%) | 2,011 (32.9%) | 2,505 (76.5%) | 3,156 (48.3%) |
| Anxiolytics | 107 (4.8%) | 76 (1.7%) | 319 (13.2%) | 217 (4.5%) | 754 (24.6%) | 599 (9.8%) | 1,213 (37.0%) | 1,375 (21.0%) |
| Antidepressants | 421 (18.9%) | 288 (6.5%) | 1,053 (43.7%) | 786 (16.4%) | 1,725 (56.2%) | 1,554 (25.4%) | 2,173 (66.4%) | 2,504 (38.3%) |
| Antipsychotics | 180 (8.1%) | 147 (3.3%) | 406 (16.9%) | 316 (6.6%) | 697 (22.7%) | 618 (10.1%) | 1,019 (31.1%) | 1,011 (15.5%) |
| **Other drug abuse (%)** | | | | | | | | |
| Heroin | (n<5) | (n<5) | 26 (1.1%) | 12 (0.2%) | 122 (4.0%) | 68 (1.1%) | 183 (5.6%) | 115 (1.8%) |
| Cocaine | 36 (1.6%) | (n<5) | 61 (2.5%) | 13 (0.3%) | 123 (4.0%) | 25 (0.4%) | 107 (3.3%) | 20 (0.3%) |
| Amphetamine | 9 (0.4%) | - | 16 (0.7%) | (n<5) | 51 (1.7%) | 12 (0.2%) | 56 (1.7%) | 14 (0.2%) |

# Supplementary Table 8: (Age subgroup analysis of incident only cohort) Odds Ratio

|  | **<20** | | **20-24** | | **25-34** | | **35<** | |
| --- | --- | --- | --- | --- | --- | --- | --- | --- |
|  | **Exposed** | **Unexposed** | **Exposed** | **Unexposed** | **Exposed** | **Unexposed** | **Exposed** | **Unexposed** |
| **All Mental Ill Health** | | | | | | | | |
| Population | 2,233 | 4,437 | 2,409 | 4,801 | 3,071 | 6,117 | 3,275 | 6,539 |
| Number of patients with condition at baseline, n (%) | 331 (14.82) | 210 (4.73) | 828 (34.37) | 548 (11.41) | 1,411 (45.95) | 1,231 (20.12) | 1,841 (56.21) | 1,997 (30.54) |
| Unadjusted Odds Ratio (95% Confidence interval) | 3.50 (2.92-4.20) | | 4.06 (3.60-4.59) | | 3.37 (3.07-3.71) | | 2.92 (2.68-3.18) | |
| p-value | p<0.01 | | p<0.01 | | p<0.01 | | p<0.01 | |
| Adjusted Odds Ratio (95% Confidence interval) | 3.75 (3.10-4.53) | | 4.18 (3.68-4.75) | | 3.44 (3.12-3.79) | | 2.86 (2.61-3.13) | |
| p-value | p<0.01 | | p<0.01 | | p<0.01 | | p<0.01 | |
| **Anxiety** | | | | | | | | |
| Population | 2,233 | 4,437 | 2,409 | 4,801 | 3,071 | 6,117 | 3,275 | 6,539 |
| Number of patients with condition at baseline, n (%) | 113 (5.06) | 95 (2.14) | 291 (12.08) | 185 (3.85) | 525 (17.10) | 466 (7.62) | 778 (23.76) | 823 (12.59) |
| Unadjusted Odds Ratio (95% Confidence interval) | 2.44 (1.85-3.22) | | 3.43 (2.83-4.15) | | 2.50 (2.19-2.86) | | 2.16 (1.94-2.41) | |
| p-value | p<0.01 | | p<0.01 | | p<0.01 | | p<0.01 | |
| Adjusted Odds Ratio (95% Confidence interval) | 2.48 (1.87-3.31) | | 3.26 (2.69-3.96) | | 2.45 (2.14-2.81) | | 2.07 (1.85-2.32) | |
| p-value | p<0.01 | | p<0.01 | | p<0.01 | | p<0.01 | |
| **Depression** | | | | | | | | |
| Population | 2,233 | 4,437 | 2,409 | 4,801 | 3,071 | 6,117 | 3,275 | 6,539 |
| Number of patients with condition at baseline, n (%) | 204 (9.14) | 122 (2.75) | 568 (23.58) | 386 (8.04) | 1,037 (33.77) | 871 (14.24) | 1,406 (42.93) | 1,466 (22.42) |
| Unadjusted Odds Ratio (95% Confidence interval) | 3.56 (2.82-4.48) | | 3.53 (3.07-4.06) | | 3.07 (2.77-3.41) | | 2.60 (2.38-2.85) | |
| p-value | p<0.01 | | p<0.01 | | p<0.01 | | p<0.01 | |
| Adjusted Odds Ratio (95% Confidence interval) | 3.75 (2.95-4.78) | | 3.61 (3.12-4.17) | | 3.14 (2.82-3.50) | | 2.55 (2.32-2.80) | |
| p-value | p<0.01 | | p<0.01 | | p<0.01 | | p<0.01 | |

|  | **<20** | | **20-24** | | **25-34** | | **35<** | |
| --- | --- | --- | --- | --- | --- | --- | --- | --- |
|  | **Exposed** | **Unexposed** | **Exposed** | **Unexposed** | **Exposed** | **Unexposed** | **Exposed** | **Unexposed** |
| **Serious Mental Illness** | | | | | | | | |
| Population | 2,233 | 4,437 | 2,409 | 4,801 | 3,071 | 6,117 | 3,275 | 6,539 |
| Number of patients with condition at baseline, n (%) | 43 (1.93) | 7 (0.16) | 116 (4.82) | 39 (0.81) | 200 (6.51) | 95 (1.55) | 365 (11.15) | 208 (3.18) |
| Unadjusted Odds Ratio (95% Confidence interval) | 12.43 (5.58-27.67) | | 6.18 (4.28-8.91) | | 4.42 (3.45-5.66) | | 3.82 (3.20-4.55) | |
| p-value | p<0.01 | | p<0.01 | | p<0.01 | | p<0.01 | |
| Adjusted Odds Ratio (95% Confidence interval) | 13.82 (6.13-31.19) | | 6.22 (4.28-9.02) | | 4.23 (3.29-5.45) | | 3.58 (2.99-4.28) | |
| p-value | p<0.01 | | p<0.01 | | p<0.01 | | p<0.01 | |
| **Combined Prescriptions** | | | | | | | | |
| Population | 2,233 | 4,437 | 2,409 | 4,801 | 3,071 | 6,117 | 3,275 | 6,539 |
| Number of patients with condition at baseline, n (%) | 566 (25.35) | 444 (10.01) | 1,281 (53.18) | 1,051 (21.89) | 2,030 (66.10) | 2,011 (32.88) | 2,505 (76.49) | 3,156 (48.26) |
| Unadjusted Odds Ratio (95% Confidence interval) | 3.05 (2.66-3.50) | | 4.05 (3.65-4.50) | | 3.98 (3.63-4.36) | | 3.49 (3.17-3.83) | |
| p-value | p<0.01 | | p<0.01 | | p<0.01 | | p<0.01 | |
| Adjusted Odds Ratio (95% Confidence interval) | 3.62 (3.13-4.20) | | 4.30 (3.85-4.80) | | 4.20 (3.82-4.63) | | 3.57 (3.23-3.94) | |
| p-value | p<0.01 | | p<0.01 | | p<0.01 | | p<0.01 | |
| **Anxiolytics** | | | | | | | | |
| Population | 2,233 | 4,437 | 2,409 | 4,801 | 3,071 | 6,117 | 3,275 | 6,539 |
| Number of patients with condition at baseline, n (%) | 107 (4.79) | 76 (1.71) | 319 (13.24) | 217 (4.52) | 754 (24.55) | 599 (9.79) | 1,213 (37.04) | 1,375 (21.03) |
| Unadjusted Odds Ratio (95% Confidence interval) | 2.89 (2.14-3.89) | | 3.22 (2.69-3.86) | | 3.00 (2.66-3.37) | | 2.21 (2.01-2.42) | |
| p-value | p<0.01 | | p<0.01 | | p<0.01 | | p<0.01 | |
| Adjusted Odds Ratio (95% Confidence interval) | 3.21 (2.36-4.37) | | 3.22 (2.68-3.87) | | 3.03 (2.68-3.42) | | 2.17 (1.97-2.39) | |
| p-value | p<0.01 | | p<0.01 | | p<0.01 | | p<0.01 | |

|  | **<20** | | **20-24** | | **25-34** | | **35<** | |  |
| --- | --- | --- | --- | --- | --- | --- | --- | --- | --- |
|  | **Exposed** | **Unexposed** | **Exposed** | **Unexposed** | **Exposed** | **Unexposed** | **Exposed** | **Unexposed** |  |
| **Antidepressants** | | | | | | | | | |
| Population | 2,233 | 4,437 | 2,409 | 4,801 | 3,071 | 6,117 | 3,275 | 6,539 | |
| Number of patients with condition at baseline, n (%) | 421 (18.85) | 288 (6.49) | 1,053 (43.71) | 786 (16.37) | 1,725 (56.17) | 1,554 (25.40) | 2,173 (66.35) | 2,504 (38.29) | |
| Unadjusted Odds Ratio (95% Confidence interval) | 3.35 (2.85-3.93) | | 3.97 (3.55-4.43) | | 3.76 (3.43-4.12) | | 3.18 (2.91-3.47) | | |
| p-value | p<0.01 | | p<0.01 | | p<0.01 | | p<0.01 | | |
| Adjusted Odds Ratio (95% Confidence interval) | 4.11 (3.45-4.89) | | 4.19 (3.73-4.71) | | 4.00 (3.63-4.40) | | 3.24 (2.95-3.56) | | |
| p-value | p<0.01 | | p<0.01 | | p<0.01 | | p<0.01 | | |
| **Antipsychotics** | | | | | | | | | |
| Population | 2,233 | 4,437 | 2,409 | 4,801 | 3,071 | 6,117 | 3,275 | 6,539 | |
| Number of patients with condition at baseline, n (%) | 180 (8.06) | 147 (3.31) | 406 (16.85) | 316 (6.58) | 697 (22.70) | 618 (10.10) | 1,019 (31.11) | 1,011 (15.46) | |
| Unadjusted Odds Ratio (95% Confidence interval) | 2.56 (2.04-3.20) | | 2.88 (2.46-3.36) | | 2.61 (2.32-2.94) | | 2.47 (2.24-2.73) | | |
| p-value | p<0.01 | | p<0.01 | | p<0.01 | | p<0.01 | | |
| Adjusted Odds Ratio (95% Confidence interval) | 2.68 (2.12-3.38) | | 2.87 (2.44-3.36) | | 2.63 (2.33-2.97) | | 2.43 (2.19-2.69) | | |
| p-value | p<0.01 | | p<0.01 | | p<0.01 | | p<0.01 | | |

# Supplementary Table 9: (Age subgroup analysis of incident only cohort) Main Results

|  | **< 20** | | **20-24** | | **25-34** | | **35 <** | |
| --- | --- | --- | --- | --- | --- | --- | --- | --- |
|  | **Exposed** | **Unexposed** | **Exposed** | **Unexposed** | **Exposed** | **Unexposed** | **Exposed** | **Unexposed** |
| **All Mental Ill Health** | | | | | | | | |
| Population | 1,902 | 4,227 | 1,581 | 4,253 | 1,660 | 4,886 | 1,434 | 4,542 |
| Outcome events, n (%) | 421 (22.13) | 312 (7.38) | 359 (22.71) | 273 (6.42) | 352 (21.20) | 334 (6.84) | 223 (15.55) | 308 (6.78) |
| Person-years | 7,780 | 16,903 | 6,072 | 15,956 | 6,703 | 18,459 | 5,433 | 18,931 |
| Crude Incidence Rate/1000 person years | 54.11 | 18.46 | 59.13 | 17.11 | 52.51 | 18.09 | 41.05 | 16.27 |
| Follow-up years, Median (IQR) | 3.04 (1.22-6.06) | 2.93 (1.14-5.94) | 2.86 (1.07-5.53) | 2.68 (1.03-5.45) | 2.95 (1.12-5.88) | 2.62 (1.08-5.54) | 2.71 (1.07-5.60) | 3.12 (1.30-5.98) |
| Unadjusted Hazard Rate ratio (95% CI) | 2.93 (2.53-3.39) | | 3.47 (2.97-4.06) | | 2.94 (2.53-3.42) | | 2.49 (2.10-2.96) | |
| p-value | p<0.01 | | p<0.01 | | p<0.01 | | p<0.01 | |
| Adjusted Hazard Rate ratio (95% CI) | 2.98 (2.56-3.46) | | 3.61 (3.08-4.24) | | 3.07 (2.63-3.58) | | 2.45 (2.05-2.93) | |
| p-value | p<0.01 | | p<0.01 | | p<0.01 | | p<0.01 | |
| **Anxiety** | | | | | | | | |
| Population | 2,120 | 4,342 | 2,118 | 4,616 | 2,546 | 5,651 | 2,497 | 5,716 |
| Outcome events, n (%) | 193 (9.10) | 121 (2.79) | 214 (10.10) | 137 (2.97) | 258 (10.13) | 196 (3.47) | 191 (7.65) | 163 (2.85) |
| Person-years | 9,769 | 18,001 | 9,221 | 17,872 | 11,386 | 22,086 | 10,380 | 23,932 |
| Crude Incidence Rate/1000 person years | 19.76 | 6.72 | 23.21 | 7.67 | 22.66 | 8.87 | 18.40 | 6.81 |
| Follow-up years, Median (IQR) | 3.54 (1.54-6.84) | 3.07 (1.19-6.24) | 3.44 (1.38-6.42) | 2.78 (1.08-5.65) | 3.47 (1.38-6.64) | 2.76 (1.13-5.78) | 3.11 (1.31-6.05) | 3.14 (1.29-6.09) |
| Unadjusted Hazard Rate ratio (95% CI) | 2.95 (2.35-3.71) | | 3.07 (2.48-3.81) | | 2.58 (2.14-3.11) | | 2.70 (2.19-3.32) | |
| p-value | p<0.01 | | p<0.01 | | p<0.01 | | p<0.01 | |
| Adjusted Hazard Rate ratio (95% CI) | 2.93 (2.33-3.70) | | 3.03 (2.44-3.76) | | 2.58 (2.13-3.11) | | 2.55 (2.06-3.16) | |
| p-value | p<0.01 | | p<0.01 | | p<0.01 | | p<0.01 | |
| **Depression** | | | | | | | | |
| Population | 2,029 | 4,315 | 1,841 | 4,415 | 2,034 | 5,246 | 1,869 | 5,073 |
| Outcome events, n (%) | 283 (13.95) | 221 (5.12) | 264 (14.34) | 191 (4.33) | 293 (14.41) | 248 (4.73) | 188 (10.06) | 259 (5.11) |
| Person-years | 8,879 | 17,510 | 7,703 | 16,771 | 8,703 | 20,228 | 7,432 | 21,084 |
| Crude Incidence Rate/1000 person years | 31.87 | 12.62 | 34.27 | 11.39 | 33.67 | 12.26 | 25.30 | 12.28 |
| Follow-up years, Median (IQR) | 3.34 (1.40-6.45) | 2.98 (1.16-6.02) | 3.25 (1.28-6.00) | 2.73 (1.05-5.52) | 3.22 (1.27-6.22) | 2.68 (1.13-5.72) | 2.94 (1.16-5.90) | 3.09 (1.28-5.98) |
| Unadjusted Hazard Rate ratio (95% CI) | 2.54 (2.13-3.03) | | 3.05 (2.53-3.68) | | 2.80 (2.36-3.32) | | 2.05 (1.70-2.47) | |
| p-value | p<0.01 | | p<0.01 | | p<0.01 | | p<0.01 | |
| Adjusted Hazard Rate ratio (95% CI) | 2.58 (2.15-3.08) | | 3.19 (2.64-3.86) | | 2.93 (2.47-3.48) | | 2.03 (1.68-2.46) | |
| p-value | p<0.01 | | p<0.01 | | p<0.01 | | p<0.01 | |

|  | **< 20** | | **20-24** | | **25-34** | | **35 <** | |  |
| --- | --- | --- | --- | --- | --- | --- | --- | --- | --- |
|  | **Exposed** | **Unexposed** | **Exposed** | **Unexposed** | **Exposed** | **Unexposed** | **Exposed** | **Unexposed** | |
| **Serious Mental Illness** | | | | | | | | | |
| Population | 2,190 | 4,430 | 2,293 | 4,762 | 2,871 | 6,022 | 2,910 | 6,331 | |
| Outcome events, n (%) | 77 (3.52) | 19 (0.43) | 82 (3.58) | 23 (0.48) | 78 (2.72) | 20 (0.33) | 63 (2.16) | 30 (0.47) | |
| Person-years | 10,479 | 18,615 | 10,686 | 18,714 | 13,816 | 24,368 | 12,742 | 26,930 | |
| Crude Incidence Rate/1000 person years | 7.35 | 1.02 | 7.67 | 1.23 | 5.65 | 0.82 | 4.94 | 1.11 | |
| Follow-up years, Median (IQR) | 3.72 (1.65-7.10) | 3.16 (1.20-6.30) | 3.76 (1.58-6.92) | 2.86 (1.12-5.72) | 3.80 (1.55-7.24) | 2.87 (1.20-6.02) | 3.35 (1.37-6.45) | 3.20 (1.31-6.20) | |
| Unadjusted Hazard Rate ratio (95% CI) | 7.44 (4.50-12.29) | | 6.65 (4.19-10.57) | | 7.27 (4.45-11.89) | | 4.45 (2.88-6.88) | | |
| p-value | p<0.01 | | p<0.01 | | p<0.01 | | p<0.01 | | |
| Adjusted Hazard Rate ratio (95% CI) | 7.39 (4.44-12.30) | | 6.63 (4.16-10.57) | | 7.02 (4.27-11.53) | | 4.08 (2.62-6.35) | | |
| p-value | p<0.01 | | p<0.01 | | p<0.01 | | p<0.01 | | |
| **Combined Prescriptions** | | | | | | | | | |
| Population | 1,667 | 3,993 | 1,128 | 3,750 | 1,041 | 4,106 | 770 | 3,383 | |
| Outcome events, n (%) | 532 (31.91) | 628 (15.73) | 356 (31.56) | 506 (13.49) | 372 (35.73) | 567 (13.81) | 232 (30.13) | 555 (16.41) | |
| Person-years | 6,192 | 14,965 | 4,116 | 13,239 | 3,733 | 14,541 | 2,625 | 13,302 | |
| Crude Incidence Rate/1000 person years | 85.91 | 41.96 | 86.49 | 38.22 | 99.64 | 38.99 | 88.38 | 41.72 | |
| Follow-up years, Median (IQR) | 2.67 (1.01-5.40) | 2.73 (1.08-5.38) | 2.68 (0.89-5.36) | 2.49 (0.96-5.14) | 2.49 (0.74-5.45) | 2.40 (0.96-5.11) | 2.30 (0.78-4.72) | 2.83 (1.19-5.62) | |
| Unadjusted Hazard Rate ratio (95% CI) | 2.05 (1.83-2.30) | | 2.29 (2.00-2.62) | | 2.58 (2.26-2.94) | | 2.09 (1.79-2.44) | | |
| p-value | p<0.01 | | p<0.01 | | p<0.01 | | p<0.01 | | |
| Adjusted Hazard Rate ratio (95% CI) | 2.22 (1.97-2.50) | | 2.40 (2.09-2.75) | | 2.64 (2.31-3.02) | | 2.19 (1.87-2.56) | | |
| p-value | p<0.01 | | p<0.01 | | p<0.01 | | p<0.01 | | |
| **Anxiolytics** | | | | | | | | | |
| Population | 2,126 | 4,361 | 2,090 | 4,584 | 2,317 | 5,518 | 2,062 | 5,164 | |
| Outcome events, n (%) | 151 (7.10) | 144 (3.30) | 183 (8.76) | 169 (3.69) | 215 (9.28) | 301 (5.45) | 217 (10.52) | 340 (6.58) | |
| Person-years | 9,886 | 17,911 | 9,241 | 17,435 | 10,376 | 21,150 | 8,365 | 21,126 | |
| Crude Incidence Rate/1000 person years | 15.27 | 8.04 | 19.80 | 9.69 | 20.72 | 14.23 | 25.94 | 16.09 | |
| Follow-up years, Median (IQR) | 3.62 (1.55-6.80) | 3.05 (1.19-6.14) | 3.49 (1.43-6.61) | 2.74 (1.08-5.50) | 3.44 (1.34-6.71) | 2.68 (1.10-5.58) | 2.92 (1.15-6.01) | 2.99 (1.22-6.00) | |
| Unadjusted Hazard Rate ratio (95% CI) | 1.93 (1.53-2.42) | | 2.10 (1.70-2.59) | | 1.50 (1.26-1.79) | | 1.61 (1.36-1.91) | | |
| p-value | p<0.01 | | p<0.01 | | p<0.01 | | p<0.01 | | |
| Adjusted Hazard Rate ratio (95% CI) | 2.03 (1.61-2.56) | | 2.10 (1.70-2.59) | | 1.49 (1.25-1.78) | | 1.64 (1.38-1.95) | | |
| p-value | p<0.01 | | p<0.01 | | p<0.01 | | p<0.01 | | |

|  | **< 20** | | **20-24** | | **25-34** | | **35 <** | |  |
| --- | --- | --- | --- | --- | --- | --- | --- | --- | --- |
|  | **Exposed** | **Unexposed** | **Exposed** | **Unexposed** | **Exposed** | **Unexposed** | **Exposed** | **Unexposed** |  |
| **Antidepressants** | | | | | | | | | |
| Population | 1,812 | 4,149 | 1,356 | 4,015 | 1,346 | 4,563 | 1,102 | 4,035 | |
| Outcome events, n (%) | 483 (26.66) | 525 (12.65) | 364 (26.84) | 437 (10.88) | 392 (29.12) | 476 (10.43) | 253 (22.96) | 528 (13.09) | |
| Person-years | 7,224 | 16,077 | 5,231 | 14,639 | 5,235 | 16,852 | 4,156 | 16,435 | |
| Crude Incidence Rate/1000 person years | 66.86 | 32.66 | 69.59 | 29.85 | 74.89 | 28.25 | 60.88 | 32.13 | |
| Follow-up years, Median (IQR) | 2.89 (1.15-5.83) | 2.81 (1.09-5.65) | 2.85 (1.01-5.64) | 2.57 (0.99-5.26) | 2.72 (0.91-5.86) | 2.57 (1.02-5.39) | 2.64 (0.95-5.46) | 2.98 (1.22-5.98) | |
| Unadjusted Hazard Rate ratio (95% CI) | 2.06 (1.82-2.33) | | 2.37 (2.06-2.72) | | 2.70 (2.36-3.09) | | 1.88 (1.62-2.18) | | |
| p-value | p<0.01 | | p<0.01 | | p<0.01 | | p<0.01 | | |
| Adjusted Hazard Rate ratio (95% CI) | 2.22 (1.96-2.52) | | 2.47 (2.14-2.84) | | 2.77 (2.42-3.17) | | 1.93 (1.65-2.25) | | |
| p-value | p<0.01 | | p<0.01 | | p<0.01 | | p<0.01 | | |
| **Antipsychotics** | | | | | | | | | |
| Population | 2,053 | 4,290 | 2,003 | 4,485 | 2,374 | 5,499 | 2,256 | 5,528 | |
| Outcome events, n (%) | 157 (7.65) | 128 (2.98) | 181 (9.04) | 139 (3.10) | 193 (8.13) | 166 (3.02) | 162 (7.18) | 216 (3.91) | |
| Person-years | 9,523 | 17,697 | 8,858 | 17,184 | 10,978 | 21,590 | 9,697 | 23,038 | |
| Crude Incidence Rate/1000 person years | 16.49 | 7.23 | 20.43 | 8.09 | 17.58 | 7.69 | 16.71 | 9.38 | |
| Follow-up years, Median (IQR) | 3.50 (1.51-6.92) | 3.06 (1.20-6.15) | 3.46 (1.39-6.51) | 2.74 (1.07-5.56) | 3.58 (1.34-7.09) | 2.76 (1.13-5.81) | 3.20 (1.26-6.36) | 3.10 (1.28-6.04) | |
| Unadjusted Hazard Rate ratio (95% CI) | 2.35 (1.86-2.97) | | 2.65 (2.12-3.30) | | 2.43 (1.97-2.99) | | 1.80 (1.47-2.21) | | |
| p-value | p<0.01 | | p<0.01 | | p<0.01 | | p<0.01 | | |
| Adjusted Hazard Rate ratio (95% CI) | 2.40 (1.89-3.05) | | 2.60 (2.08-3.25) | | 2.45 (1.99-3.03) | | 1.85 (1.50-2.28) | | |
| p-value | p<0.01 | | p<0.01 | | p<0.01 | | p<0.01 | | |

# Supplementary Table 10: Gender subgroup analysis of whole cohort

| **Males** | **All Mental Ill Health** | | **Anxiety** | | **Depression** | | **Serious Mental Illness** | |
| --- | --- | --- | --- | --- | --- | --- | --- | --- |
|  | **Exposed** | **Unexposed** | **Exposed** | **Unexposed** | **Exposed** | **Unexposed** | **Exposed** | **Unexposed** |
| Population | 12,010 | 35,890 | 18,241 | 40,375 | 15,178 | 38,200 | 19,384 | 42,486 |
| Outcome events, n(%) | 1,932 (16.09) | 2,027 (5.65) | 1,218 (6.68) | 994 (2.46) | 1,461 (9.63) | 1,498 (3.92) | 456 (2.35) | 144 (0.34) |
| Person-years | 45,160 | 138,757 | 72,755 | 158,455 | 58,973 | 149,133 | 80,508 | 169,818 |
| Crude Incidence Rate/1000 person years | 42.78 | 14.61 | 16.74 | 6.27 | 24.77 | 10.04 | 5.66 | 0.85 |
| Follow-up years, Median(IQR) | 2.64 (1.01-5.51) | 2.82 (1.13-5.67) | 2.88 (1.12-5.82) | 2.88 (1.15-5.78) | 2.79 (1.08-5.67) | 2.85 (1.15-5.74) | 3.01 (1.18-6.07) | 2.93 (1.18-5.89) |
| Unadjusted Hazard Rate ratio (95% CI) | 2.93 (2.75-3.11) | | 2.67 (2.46-2.91) | | 2.47 (2.30-2.66) | | 6.76 (5.61-8.16) | |
| p-value | p<0.01 | | p<0.01 | | p<0.01 | | p<0.01 | |
| Adjusted Hazard Rate ratio (95% CI) | 2.80 (2.63-2.98) | | 2.55 (2.34-2.77) | | 2.40 (2.23-2.58) | | 6.45 (5.34-7.79) | |
| p-value | p<0.01 | | p<0.01 | | p<0.01 | | p<0.01 | |

| **Males** | **Combined Prescriptions** | | **Anxiolytics** | | **Antidepressants** | | **Antipsychotics** | |
| --- | --- | --- | --- | --- | --- | --- | --- | --- |
|  | **Exposed** | **Unexposed** | **Exposed** | **Unexposed** | **Exposed** | **Unexposed** | **Exposed** | **Unexposed** |
| Population | 10,122 | 30,816 | 17,845 | 39,044 | 12,352 | 33,858 | 17,800 | 39,749 |
| Outcome events, n(%) | 2,742 (27.09) | 4,036 (13.10) | 1,428 (8.00) | 1,655 (4.24) | 2,675 (21.66) | 3,416 (10.09) | 1,118 (6.28) | 1,016 (2.56) |
| Person-years | 33,310 | 112,624 | 69,234 | 150,691 | 43,388 | 127,944 | 70,878 | 156,052 |
| Crude Incidence Rate/1000 person years | 82.32 | 35.84 | 20.63 | 10.98 | 61.65 | 26.70 | 15.77 | 6.51 |
| Follow-up years, Median(IQR) | 2.19 (0.79-4.77) | 2.59 (1.03-5.32) | 2.72 (1.03-5.63) | 2.80 (1.12-5.65) | 2.36 (0.88-5.14) | 2.70 (1.07-5.52) | 2.81 (1.07-5.81) | 2.87 (1.15-5.76) |
| Unadjusted Hazard Rate ratio (95% CI) | 2.28 (2.17-2.39) | | 1.89 (1.76-2.03) | | 2.30 (2.18-2.42) | | 2.46 (2.26-2.68) | |
| p-value | p<0.01 | | p<0.01 | | p<0.01 | | p<0.01 | |
| Adjusted Hazard Rate ratio (95% CI) | 2.30 (2.19-2.41) | | 1.90 (1.77-2.04) | | 2.29 (2.17-2.41) | | 2.42 (2.22-2.64) | |
| p-value | p<0.01 | | p<0.01 | | p<0.01 | | p<0.01 | |

| **Females** | **All Mental Ill Health** | | **Anxiety** | | **Depression** | | **Serious Mental Illness** | |
| --- | --- | --- | --- | --- | --- | --- | --- | --- |
|  | **Exposed** | **Unexposed** | **Exposed** | **Unexposed** | **Exposed** | **Unexposed** | **Exposed** | **Unexposed** |
| Population | 2,553 | 9,273 | 4,945 | 11,497 | 3,350 | 10,050 | 5,852 | 12,748 |
| Outcome events, n(%) | 547 (21.43) | 937 (10.10) | 471 (9.52) | 529 (4.60) | 461 (13.76) | 729 (7.25) | 125 (2.14) | 43 (0.34) |
| Person-years | 7,331 | 32,993 | 16,515 | 43,352 | 10,433 | 36,709 | 21,496 | 50,064 |
| Crude Incidence Rate/1000 person years | 74.62 | 28.40 | 28.52 | 12.20 | 44.19 | 19.86 | 5.81 | 0.86 |
| Follow-up years, Median(IQR) | 1.84 (0.69-3.93) | 2.58 (1.02-5.14) | 2.27 (0.85-4.77) | 2.79 (1.11-5.49) | 2.05 (0.77-4.24) | 2.66 (1.05-5.31) | 2.51 (0.98-5.31) | 2.93 (1.20-5.82) |
| Unadjusted Hazard Rate ratio (95% CI) | 2.58 (2.32-2.87) | | 2.32 (2.05-2.63) | | 2.19 (1.95-2.47) | | 6.78 (4.79-9.58) | |
| p-value | p<0.01 | | p<0.01 | | p<0.01 | | p<0.01 | |
| Adjusted Hazard Rate ratio (95% CI) | 2.56 (2.30-2.85) | | 2.28 (2.01-2.59) | | 2.20 (1.95-2.48) | | 6.32 (4.45-8.99) | |
| p-value | p<0.01 | | p<0.01 | | p<0.01 | | p<0.01 | |

| **Females** | **Combined Prescriptions** | | **Anxiolytics** | | **Antidepressants** | | **Antipsychotics** | |
| --- | --- | --- | --- | --- | --- | --- | --- | --- |
|  | **Exposed** | **Unexposed** | **Exposed** | **Unexposed** | **Exposed** | **Unexposed** | **Exposed** | **Unexposed** |
| Population | 2,011 | 7,007 | 4,832 | 10,944 | 2,446 | 8,040 | 4,775 | 10,878 |
| Outcome events, n(%) | 729 (36.25) | 1,483 (21.16) | 511 (10.58) | 773 (7.06) | 754 (30.83) | 1,350 (16.79) | 444 (9.30) | 660 (6.07) |
| Person-years | 4,859 | 21,845 | 15,708 | 40,228 | 6,621 | 26,943 | 15,939 | 40,196 |
| Crude Incidence Rate/1000 person years | 150.02 | 67.89 | 32.53 | 19.22 | 113.88 | 50.11 | 27.86 | 16.42 |
| Follow-up years, Median(IQR) | 1.42 (0.51-3.25) | 2.16 (0.84-4.40) | 2.08 (0.76-4.64) | 2.68 (1.07-5.32) | 1.62 (0.59-3.65) | 2.35 (0.91-4.79) | 2.17 (0.83-4.74) | 2.70 (1.08-5.35) |
| Unadjusted Hazard Rate ratio (95% CI) | 2.16 (1.98-2.37) | | 1.68 (1.50-1.88) | | 2.23 (2.04-2.44) | | 1.69 (1.50-1.91) | |
| p-value | p<0.01 | | p<0.01 | | p<0.01 | | p<0.01 | |
| Adjusted Hazard Rate ratio (95% CI) | 2.18 (1.99-2.38) | | 1.71 (1.52-1.91) | | 2.24 (2.05-2.46) | | 1.67 (1.47-1.89) | |
| p-value | p<0.01 | | p<0.01 | | p<0.01 | | p<0.01 | |

# Supplementary Table 11: Gender subgroup analysis of incidence cohort only

| **Males** | **All Mental Ill Health** | | **Anxiety** | | **Depression** | | **Serious Mental Illness** | |
| --- | --- | --- | --- | --- | --- | --- | --- | --- |
|  | **Exposed** | **Unexposed** | **Exposed** | **Unexposed** | **Exposed** | **Unexposed** | **Exposed** | **Unexposed** |
| Population | 5,555 | 14,599 | 7,501 | 16,245 | 6,519 | 15,460 | 8,113 | 17,029 |
| Outcome events, n(%) | 1,080 (19.44) | 886 (6.07) | 627 (8.36) | 431 (2.65) | 798 (12.24) | 646 (4.18) | 233 (2.87) | 71 (0.42) |
| Person-years | 22,839 | 58,052 | 33,922 | 65,944 | 28,307 | 62,131 | 38,428 | 70,317 |
| Crude Incidence Rate/1000 person years | 47.29 | 15.26 | 18.48 | 6.54 | 28.19 | 10.40 | 6.06 | 1.01 |
| Follow-up years, Median(IQR) | 3.08 (1.20-6.02) | 2.89 (1.17-5.79) | 3.54 (1.48-6.69) | 2.97 (1.20-5.98) | 3.34 (1.34-6.39) | 2.93 (1.20-5.89) | 3.77 (1.58-7.04) | 3.03 (1.24-6.08) |
| Unadjusted Hazard Rate ratio (95% CI) | 3.11 (2.85-3.40) | | 2.85 (2.53-3.23) | | 2.74 (2.47-3.04) | | 6.24 (4.78-8.14) | |
| p-value | p<0.01 | | p<0.01 | | p<0.01 | | p<0.01 | |
| Adjusted Hazard Rate ratio (95% CI) | 2.98 (2.72-3.26) | | 2.72 (2.40-3.08) | | 2.65 (2.39-2.94) | | 5.94 (4.54-7.76) | |
| p-value | p<0.01 | | p<0.01 | | p<0.01 | | p<0.01 | |

| **Males** | **Combined Prescriptions** | | **Anxiolytics** | | **Antidepressants** | | **Antipsychotics** | |
| --- | --- | --- | --- | --- | --- | --- | --- | --- |
|  | **Exposed** | **Unexposed** | **Exposed** | **Unexposed** | **Exposed** | **Unexposed** | **Exposed** | **Unexposed** |
| Population | 4,042 | 12,696 | 7,034 | 15,747 | 4,912 | 13,862 | 7,115 | 15,985 |
| Outcome events, n(%) | 1,261 (31.20) | 1,706 (13.44) | 598 (8.50) | 671 (4.26) | 1,249 (25.43) | 1,460 (10.53) | 518 (7.28) | 410 (2.56) |
| Person-years | 15,066 | 47,884 | 31,602 | 62,896 | 19,554 | 53,961 | 32,765 | 64,871 |
| Crude Incidence Rate/1000 person years | 83.70 | 35.63 | 18.92 | 10.67 | 63.87 | 27.06 | 15.81 | 6.32 |
| Follow-up years, Median(IQR) | 2.67 (0.94-5.48) | 2.67 (1.08-5.46) | 3.48 (1.40-6.67) | 2.91 (1.17-5.86) | 2.92 (1.06-5.86) | 2.80 (1.12-5.67) | 3.58 (1.46-6.88) | 2.96 (1.21-5.97) |
| Unadjusted Hazard Rate ratio (95% CI) | 2.36 (2.19-2.53) | | 1.82 (1.63-2.03) | | 2.38 (2.20-2.56) | | 2.61 (2.29-2.97) | |
| p-value | p<0.01 | | p<0.01 | | p<0.01 | | p<0.01 | |
| Adjusted Hazard Rate ratio (95% CI) | 2.39 (2.22-2.57) | | 1.83 (1.63-2.04) | | 2.38 (2.20-2.56) | | 2.57 (2.25-2.93) | |
| p-value | p<0.01 | | p<0.01 | | p<0.01 | | p<0.01 | |

| **Females** | **All Mental Ill Health** | | **Anxiety** | | **Depression** | | **Serious Mental Illness** | |
| --- | --- | --- | --- | --- | --- | --- | --- | --- |
|  | **Exposed** | **Unexposed** | **Exposed** | **Unexposed** | **Exposed** | **Unexposed** | **Exposed** | **Unexposed** |
| Population | 1,022 | 3,309 | 1,780 | 4,080 | 1,254 | 3,589 | 2,151 | 4,516 |
| Outcome events, n(%) | 275 (26.91) | 341 (10.31) | 229 (12.87) | 186 (4.56) | 230 (18.34) | 273 (7.61) | 67 (3.11) | 21 (0.47) |
| Person-years | 3,149 | 12,196 | 6,834 | 15,947 | 4,410 | 13,462 | 9,296 | 18,311 |
| Crude Incidence Rate/1000 person years | 87.33 | 27.96 | 33.51 | 11.66 | 52.16 | 20.28 | 7.21 | 1.15 |
| Follow-up years, Median(IQR) | 2.04 (0.84-4.28) | 2.59 (1.01-5.33) | 2.83 (1.11-5.57) | 2.76 (1.08-5.80) | 2.39 (0.98-4.91) | 2.64 (1.04-5.45) | 3.20 (1.29-6.34) | 2.92 (1.14-6.03) |
| Unadjusted Hazard Rate ratio (95% CI) | 3.07 (2.62-3.60) | | 2.87 (2.37-3.48) | | 2.55 (2.14-3.04) | | 6.40 (3.92-10.46) | |
| p-value | p<0.01 | | p<0.01 | | p<0.01 | | p<0.01 | |
| Adjusted Hazard Rate ratio (95% CI) | 3.04 (2.58-3.58) | | 2.77 (2.27-3.37) | | 2.59 (2.16-3.10) | | 6.12 (3.72-10.07) | |
| p-value | p<0.01 | | p<0.01 | | p<0.01 | | p<0.01 | |

| **Females** | **Combined Prescriptions** | | **Anxiolytics** | | **Antidepressants** | | **Antipsychotics** | |
| --- | --- | --- | --- | --- | --- | --- | --- | --- |
|  | **Exposed** | **Unexposed** | **Exposed** | **Unexposed** | **Exposed** | **Unexposed** | **Exposed** | **Unexposed** |
| Population | 564 | 2,536 | 1,561 | 3,880 | 704 | 2,900 | 1,571 | 3,817 |
| Outcome events, n(%) | 231 (40.96) | 550 (21.69) | 168 (10.76) | 283 (7.29) | 243 (34.52) | 506 (17.45) | 175 (11.14) | 239 (6.26) |
| Person-years | 1,601 | 8,164 | 6,266 | 14,725 | 2,291 | 10,042 | 6,290 | 14,638 |
| Crude Incidence Rate/1000 person years | 144.28 | 67.37 | 26.81 | 19.22 | 106.08 | 50.39 | 27.82 | 16.33 |
| Follow-up years, Median(IQR) | 1.80 (0.60-4.03) | 2.22 (0.84-4.48) | 2.86 (1.11-6.01) | 2.70 (1.03-5.50) | 2.21 (0.78-4.67) | 2.38 (0.90-4.97) | 2.83 (1.11-5.92) | 2.72 (1.05-5.51) |
| Unadjusted Hazard Rate ratio (95% CI) | 2.12 (1.82-2.47) | | 1.41 (1.16-1.70) | | 2.10 (1.80-2.45) | | 1.72 (1.42-2.10) | |
| p-value | p<0.01 | | p<0.01 | | p<0.01 | | p<0.01 | |
| Adjusted Hazard Rate ratio (95% CI) | 2.13 (1.81-2.49) | | 1.46 (1.21-1.78) | | 2.15 (1.84-2.52) | | 1.72 (1.41-2.10) | |
| p-value | p<0.01 | | p<0.01 | | p<0.01 | | p<0.01 | |

#

# STROBE checklist

|  | Item No | Recommendation | Reporting Location |  |
| --- | --- | --- | --- | --- |
| **Title and abstract** | 1 | (*a*) Indicate the study’s design with a commonly used term in the title or the abstract | Abstract |  |
|  |  | (*b*) Provide in the abstract an informative and balanced summary of what was done and what was found | Abstract |  |
| Introduction | | |  |  |
| Background/rationale | 2 | Explain the scientific background and rationale for the investigation being reported | Introduction |  |
| Objectives | 3 | State specific objectives, including any prespecified hypotheses | Introduction |  |
| Methods | | |  |  |
| Study design | 4 | Present key elements of study design early in the paper | Methods |  |
| Setting | 5 | Describe the setting, locations, and relevant dates, including periods of recruitment, exposure, follow-up, and data collection | Methods |  |
| Participants | 6 | (*a*) Give the eligibility criteria, and the sources and methods of selection of participants. Describe methods of follow-up | Methods |  |
|  |  | (*b*) For matched studies, give matching criteria and number of exposed and unexposed | Methods  Results: table 1 |  |
| Variables | 7 | Clearly define all outcomes, exposures, predictors, potential confounders, and effect modifiers. Give diagnostic criteria, if applicable | Methods |  |
| Data sources/ measurement | 8* | For each variable of interest, give sources of data and details of methods of assessment (measurement). Describe comparability of assessment methods if there is more than one group | Methods |  |
| Bias | 9 | Describe any efforts to address potential sources of bias | Methods/discussion |  |
| Study size | 10 | Explain how the study size was arrived at | Methods |  |
| Quantitative variables | 11 | Explain how quantitative variables were handled in the analyses. If applicable, describe which groupings were chosen and why | Methods |  |
| Statistical methods | 12 | (*a*) Describe all statistical methods, including those used to control for confounding | Methods |  |
|  |  | (*b*) Describe any methods used to examine subgroups and interactions | N/A |  |
|  |  | (*c*) Explain how missing data were addressed | Methods |  |
|  |  | (*d*) If applicable, explain how loss to follow-up was addressed | Methods |  |
|  |  | (*e*) Describe any sensitivity analyses | Methods |  |
| Results | | |  |  |
| Participants | 13* | (a) Report numbers of individuals at each stage of study—eg numbers potentially eligible, examined for eligibility, confirmed eligible, included in the study, completing follow-up, and analysed | Methods and results |  |
|  |  | (b) Give reasons for non-participation at each stage | N/A |  |
|  |  | (c) Consider use of a flow diagram | N/A |  |
| Descriptive data | 14* | (a) Give characteristics of study participants (eg demographic, clinical, social) and information on exposures and potential confounders | Results |  |
|  |  | (b) Indicate number of participants with missing data for each variable of interest | Table 1 |  |
|  |  | (c) Summarise follow-up time (eg, average and total amount) | Table 1 |  |
| Outcome data | 15* | Report numbers of outcome events or summary measures over time |  |  |
| Main results | 16 | (*a*) Give unadjusted estimates and, if applicable, confounder-adjusted estimates and their precision (eg, 95% confidence interval). Make clear which confounders were adjusted for and why they were included | Results and Table 2,3 |  |
|  |  | (*b*) Report category boundaries when continuous variables were categorized | Results and Table 2,3 |  |
|  |  | (*c*) If relevant, consider translating estimates of relative risk into absolute risk for a meaningful time period | N/A |  |
| Other analyses | 17 | Report other analyses done—eg analyses of subgroups and interactions, and sensitivity analyses | Mentioned in results and throughout appendices |  |
| Discussion | | |  |  |
| Key results | 18 | Summarise key results with reference to study objectives | Discussion |  |
| Limitations | 19 | Discuss limitations of the study, taking into account sources of potential bias or imprecision. Discuss both direction and magnitude of any potential bias | Discussion |  |
| Interpretation | 20 | Give a cautious overall interpretation of results considering objectives, limitations, multiplicity of analyses, results from similar studies, and other relevant evidence | Discussion |  |
| Generalisability | 21 | Discuss the generalisability (external validity) of the study results | Discussion |  |
| Other information | | |  |  |
| Funding | 22 | Give the source of funding and the role of the funders for the present study and, if applicable, for the original study on which the present article is based | None declared |  |
